# Supplementary material for: Axon-Axon Interactions Regulate Topographic Optic Tract Sorting via CYFIP2-Dependent WAVE Complex Function
Source: Neuron. 2018 Mar 7;97(5):1078–1093.e6. doi: 10.1016/j.neuron.2018.01.027 (PMC5855093; doi:10.1016/j.neuron.2018.01.027)
Supplement: Document S1. Figures S1–S4 [file mmc1.pdf]

Neuron, Volume *97*

Supplemental Information

Axon-Axon Interactions Regulate

Topographic Optic Tract Sorting

via CYFIP2-Dependent WAVE Complex Function

Jean-Michel Cioni, Hovy Ho-Wai Wong, Dario Bressan, Lay Kodama, William A. Harris, and Christine E. Holt

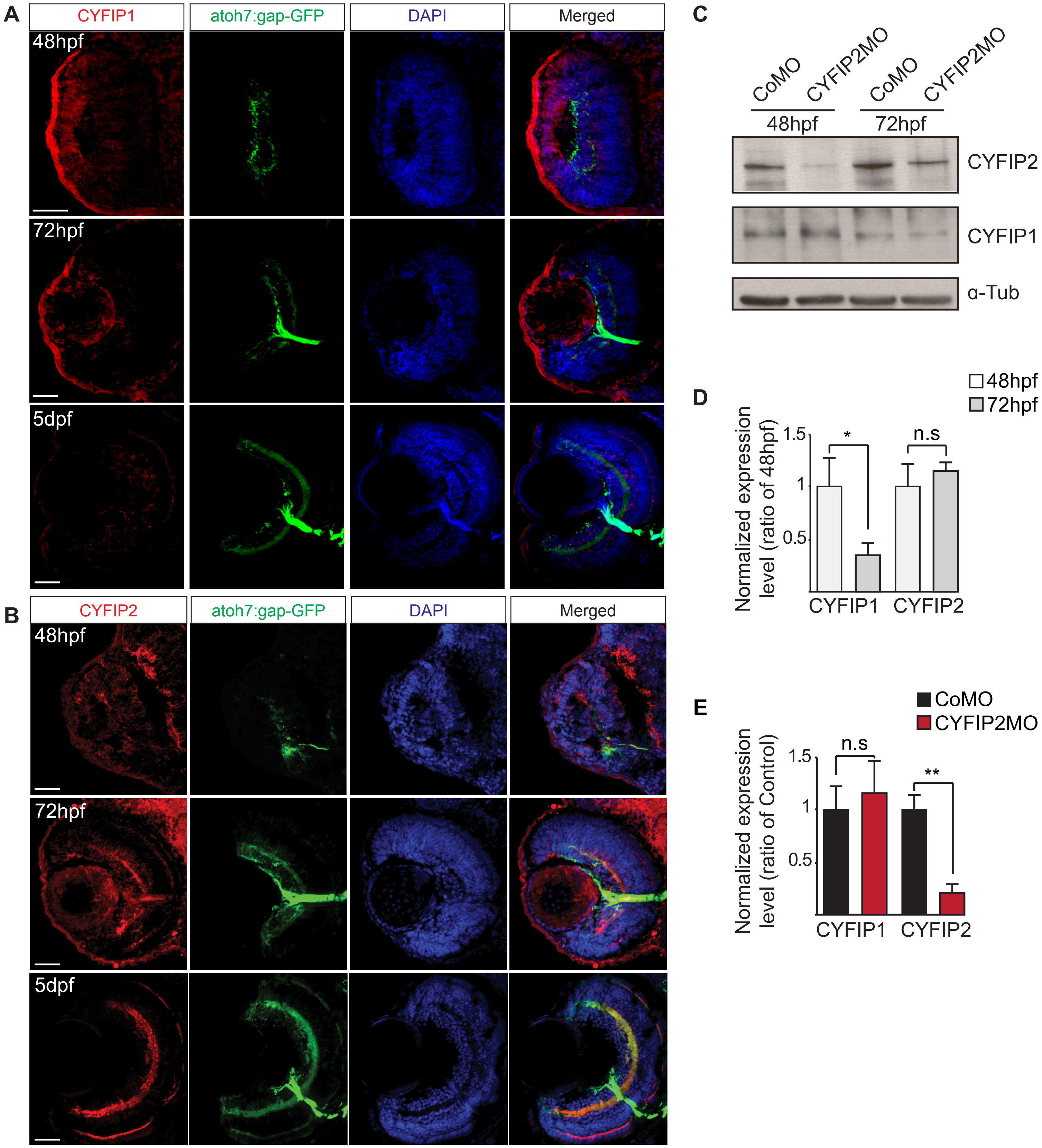
A
CYFIP1
atoh7:gap-GFP
DAPI
Merged
48hpf
72hpf
5dpf
B
CYFIP2
atoh7:gap-GFP
DAPI
Merged
48hpf
72hpf
5dpf
C
CoMO
CYFIP2MO
CoMO
CYFIP2MO
48hpf
72hpf
CYFIP2
CYFIP1
α-Tub
D
48hpf
72hpf
Normalized expression level (ratio of 48hpf)
1.5
1
0.5
*
n.s
CYFIP1
CYFIP2
E
CoMO
CYFIP2MO
Normalized expression level (ratio of Control)
1.5
1
0.5
n.s
**
CYFIP1
CYFIP2

FIGURE S1

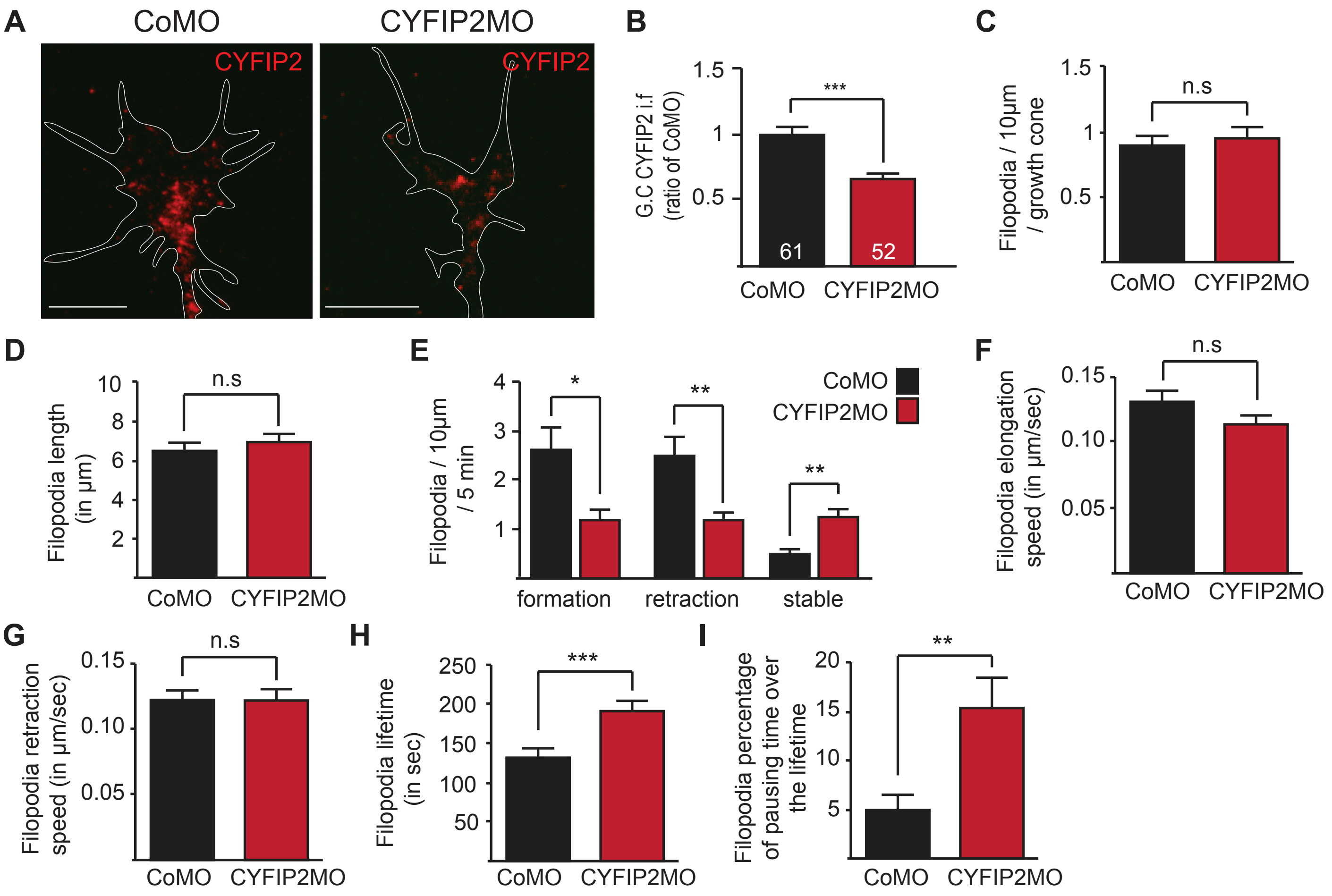
A
CoMO
CYFIP2MO
CYFIP2
CYFIP2
B
G.C CYFIP2 i.f (ratio of CoMO)
1.5
1
0.5
***
61
52
CoMO
CYFIP2MO
C
Filopodia / 10µm / growth cone
1.5
1
0.5
n.s
CoMO
CYFIP2MO
D
Filopodia length (in µm)
10
8
6
4
2
n.s
CoMO
CYFIP2MO
E
Filopodia / 10µm / 5 min
4
3
2
1
*
**
**
CoMO
CYFIP2MO
formation
retraction
stable
F
Filopodia elongation speed (in µm/sec)
0.15
0.10
0.05
n.s
CoMO
CYFIP2MO
G
Filopodia retraction speed (in µm/sec)
0.15
0.10
0.05
n.s
CoMO
CYFIP2MO
H
Filopodia lifetime (in sec)
250
200
150
100
50
***
CoMO
CYFIP2MO
I
Filopodia percentage of pausing time over the lifetime
20
15
10
5
**
CoMO
CYFIP2MO

FIGURE S2

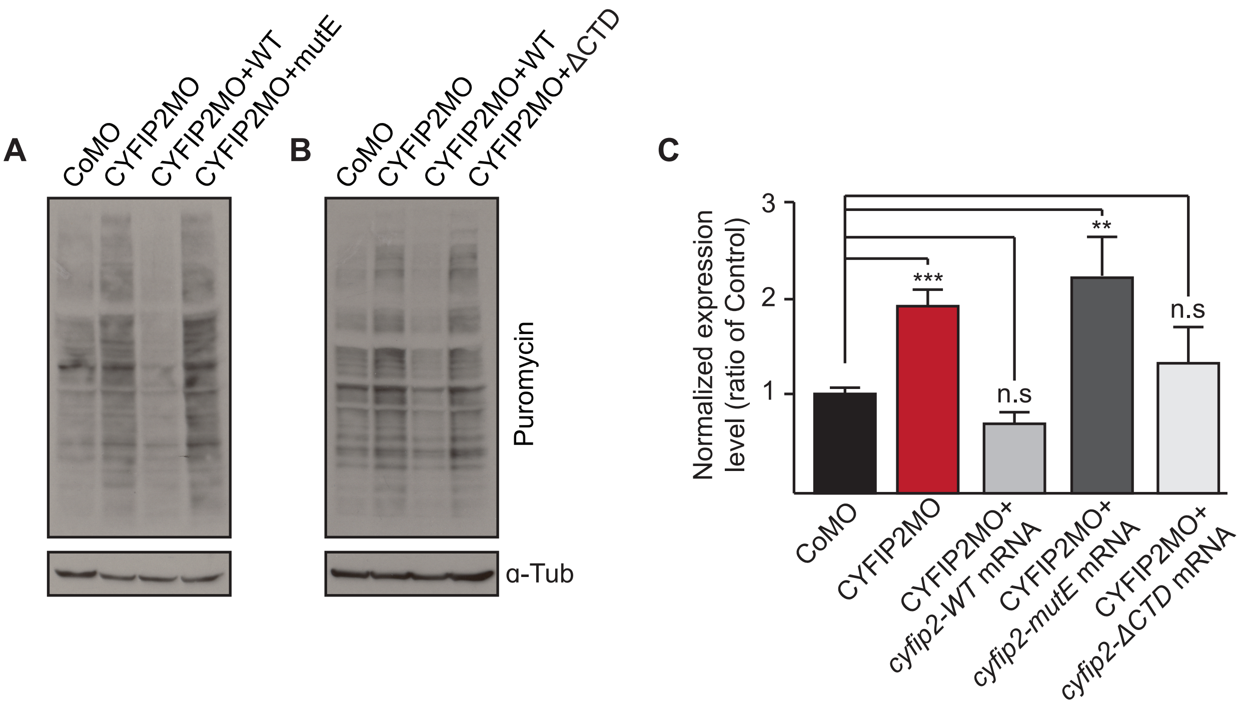
A
CoMO
CYFIP2MO
CYFIP2MO+WT
CYFIP2MO+mutE
B
CoMO
CYFIP2MO
CYFIP2MO+WT
CYFIP2MO+ΔCTD
Puromycin
α-Tub
C
Normalized expression level (ratio of Control)
3
2
1
***
n.s
**
n.s
CoMO
CYFIP2MO
CYFIP2MO+ cyfip2-WT mRNA
CYFIP2MO+ cyfip2-mutE mRNA
CYFIP2MO+ cyfip2-ΔCTD mRNA

FIGURE S3

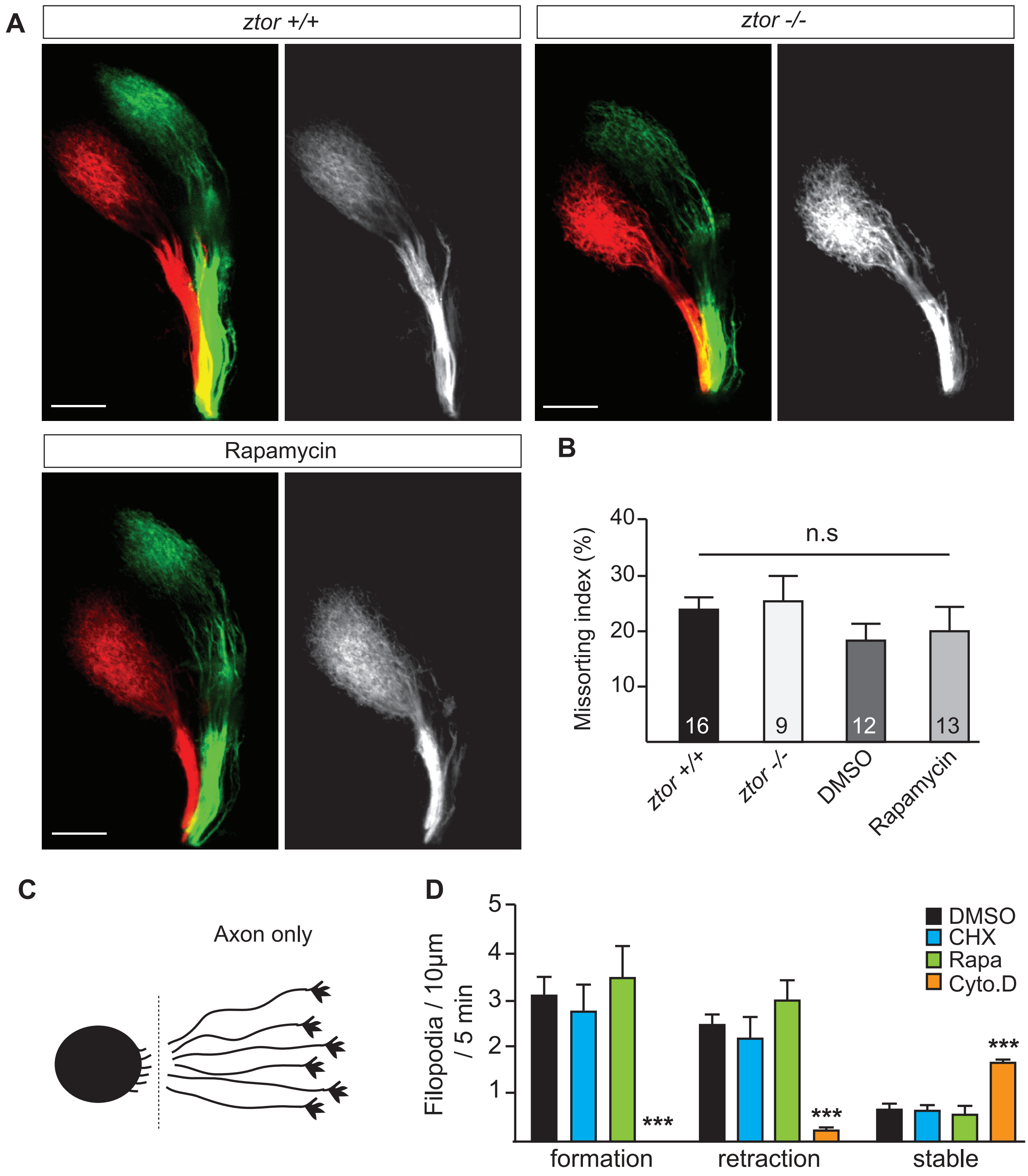

A
ztor +/+
ztor -/-
Rapamycin
B
Missorting index (%)
40
30
20
10
n.s
16
9
12
13
ztor +/+
ztor -/-
DMSO
Rapamycin
C
Axon only
D
Filopodia / 10µm / 5 min
5
4
3
2
1
DMSO
CHX
Rapa
Cyto.D
***
***
***
formation
retraction
stable

FIGURE S4

Supplementary information

Figure S1: Related to Figure 1. CYFIP1 and CYFIP2 expression during early zebrafish retina development.

(A) Representative immunostainings of CYFIP1 and DAPI at 48hpf, 72hpf and 5dpf on retina slices from atoh7:gap-GFP zebrafish line (n=8 zebrafish for each developmental stage). Scale bars=50µm. (B) Representative immunostainings of CYFIP2 and DAPI at 48hpf, 72hpf and 5dpf on retina slices from atoh7:gap-GFP zebrafish line (n=12 zebrafish for each developmental stage). Scale bars=50µm. (C-E) Representative western blots for CYFIP1 and CYFIP2 on zebrafish heads lysates at 48hpf and 72hpf for CoMO- and CYFIP2MO-injected embryos. (D) Quantifications of the signal obtained for CYFIP1 and CYFIP2 at 48hpf compared to 72hpf (n=4, normalized to α-Tubulin, Mann Whitney test). (E) Quantifications of the signal obtained for CYFIP1 and CYFIP2 at 48hpf in CoMO and CYFIP2MO conditions (n=4, normalized to α-Tubulin). Error bars represent SEM. *$p<0.05$, **$p<0.01$, n.s: non-significant (Mann Whitney test for D and E).

Figure S2: Related to Figure 4. CYFIP2 regulates the dynamic behaviors of filopodia in RGC growth cones.

(A) Representative CYFIP2 immunostainings on stage 32 Xenopus retinal growth cone (GC) in CoMO and CYFIP2MO conditions. (B) Quantification of CYFIP2 signal intensity in GC of CYFIP2MO- compared to CoMO-injected embryos. Number of axons analyzed is indicated on the graph. (C, D) Quantifications of the number (C) and length (D) of GC filopodia in CYFIP2MO (n=18 GC, n=101 filopodia) compared to CoMO (CoMO: n=16 GC, n=80 filopodia) conditions. (E) Quantifications of the frequency of formation, retraction and stable filopodia in GCs from CYFIP2MO-injected (n=17 GC) compared to CoMO-injected (n=15 GCs) retina explants over 5min recording. (F) Quantifications of the speed during active filopodia elongation in CYFIP2MO (n=15 filopodia) compared to CoMO (n=15 filopodia) conditions. (G) Quantifications of the speed during active filopodia retraction in CYFIP2MO

(n=13 filopodia) compared to CoMO (n=10 filopodia) conditions. (H) Quantifications of filopodia lifetime in CYFIP2MO (n=13 GC, n=97 filopodia) compared to CoMO (n=11 GC, n=111 filopodia) conditions. (I) Percentage of the time spent pausing during the filopodia lifetime. Error bars represent SEM. * p<0.05, ** p<0.01, *** p<0.001, n.s: non-significant (Mann Whitney test for B-I). Scale bars 5μm.

Figure S3: Related to Figure 7. CYFIP2 exerts a translational control *in vivo*

(A-B) Representative western blots showing puromycin incorporation in 48hpf zebrafish embryos for the indicated conditions. (C) Quantification of the signal obtained for puromycin normalized to α-Tubulin. Co-injection of CYFIP2MO + *CYFIP2WT* (n=7experiments) or *CYFIP2ΔCTD* (n=4 experiments), but not *CYFIP2mutE* (n=5 experiments), mRNAs rescue the increase in puromycin signal observed in CYFIP2MO-inected embryos (n=7 experiments) compared to CoMO (n=7 experiments). Error bars represent SEM. ** p<0.01, *** p<0.001, n.s: non-significant (Mann Whitney test).

Figure S4: Related to Figure 7. Translation regulation is not required for proper growth cone filopodial dynamics and D-V axon sorting.

(A) Dorsal and Ventral RGC axons were labeled by respective injections of DiI (red) and DiO (green) fluorescent dyes in zebrafish embryos retina at 5dpf. Scale bars: 50 μm. (B) The missorting index (MI) was quantified as the ratio of the intensity signal of the missorted D (Dm) axons to all the D axons (Dm+Ds) (Mann Whitney test, n.s: non significant). The number of zebrafish analyzed is indicated on the bars. (C) Schematic illustrating the axon-only culture used for the analysis of filopodia dynamics. (D) Quantifications of the frequency of formation, retraction and stable filopodia in growth cones of axon-only explants treated with DMSO (n=7 GC), cycloheximide (CHX) (n=7 GC), rapamycin (rapa) (n=6 GC) and cytochalasin D (cyto.D) (n=7 GC). Error bars represent SEM. *** p<0.001 (Mann Whitney test for B and D).
