# Supplementary material for: Axon-Axon Interactions Regulate Topographic Optic Tract Sorting via CYFIP2-Dependent WAVE Complex Function
Source: Neuron. 2018 Mar 7;97(5):1078–1093.e6. doi: 10.1016/j.neuron.2018.01.027 (PMC5855093; doi:10.1016/j.neuron.2018.01.027)
Supplement: Document S2. Article plus Supplemental Information [file mmc8.pdf]

Article

Neuron

Axon-Axon Interactions Regulate Topographic Optic Tract Sorting via CYFIP2-Dependent WAVE Complex Function

Highlights

- CYFIP1 and CYFIP2 serve non-redundant functions in retinal axon growth and guidance
- CYFIP2 regulates growth cone filopodial dynamics and axon-axon responses
- CYFIP2 interacts with RNPs and the WRC in distinct cellular compartments
- Axon sorting is mediated by CYFIP2's interaction with the WRC

Authors

Jean-Michel Cioni,
Hovy Ho-Wai Wong, Dario Bressan,
Lay Kodama, William A. Harris,
Christine E. Holt

Correspondence

ceh33@cam.ac.uk

In Brief

Axon sorting within nerve tracts is important for the establishment of neural connectivity in the vertebrate nervous system. In this paper, Cioni et al. reveal new insights on how retinal axons segregate in the optic tract through CYFIP2-mediated axon-axon signaling.

Cioni et al., 2018, Neuron *97*, 1078–1093
March 7, 2018 © 2018 The Authors. Published by Elsevier Inc.
https://doi.org/10.1016/j.neuron.2018.01.027

Axon-Axon Interactions Regulate Topographic Optic Tract Sorting via CYFIP2-Dependent WAVE Complex Function

Jean-Michel Cioni,[1] Hovy Ho-Wai Wong,[1] Dario Bressan,[2] Lay Kodama,[1] William A. Harris,[1] and Christine E. Holt[1,3,*]
[1]Department of Physiology, Development and Neuroscience, University of Cambridge, Downing Street, Cambridge, CB2 3DY, UK
[2]Cancer Research UK Cambridge Institute, University of Cambridge, Li Ka Shing Centre, Robinson Way, Cambridge, CB2 0RE, UK
[3]Lead Contact
*Correspondence: ceh33@cam.ac.uk
https://doi.org/10.1016/j.neuron.2018.01.027

SUMMARY

The axons of retinal ganglion cells (RGCs) are topographically sorted before they arrive at the optic tectum. This pre-target sorting, typical of axon tracts throughout the brain, is poorly understood. Here, we show that cytoplasmic FMR1-interacting proteins (CYFIPs) fulfill non-redundant functions in RGCs, with CYFIP1 mediating axon growth and CYFIP2 specifically involved in axon sorting. We find that CYFIP2 mediates homotypic and heterotypic contact-triggered fasciculation and repulsion responses between dorsal and ventral axons. CYFIP2 associates with transporting ribonucleoprotein particles in axons and regulates translation. Axon-axon contact stimulates CYFIP2 to move into growth cones where it joins the actin nucleating WAVE regulatory complex (WRC) in the periphery and regulates actin remodeling and filopodial dynamics. CYFIP2's function in axon sorting is mediated by its binding to the WRC but not its translational regulation. Together, these findings uncover CYFIP2 as a key regulatory link between axon-axon interactions, filopodial dynamics, and optic tract sorting.

INTRODUCTION

In the early 1940s, Roger Sperry proposed a chemoaffinity hypothesis to explain the precise topographic mapping of the retina onto the optic tectum (superior colliculus) (Sperry, 1963). Later studies showed that this chemoaffinity mechanism is mediated by the graded expression of ephrin ligands in the tectum and their respective Eph receptors in RGC axons (Feldheim and O'Leary, 2010; McLaughlin et al., 2003). However, it has been observed in several systems that even before arriving at the tectum, retinal axons are sorted topographically within the optic tract (OT) and that this pre-target sorting is critical for the formation of accurate retinotectal maps (Plas et al., 2005; Scholes, 1979; Stuermer, 1988). Many other regions of the brain are also interconnected by topographically ordered tracts, and recent observations strongly suggest that sorting within such tracts depends on axon-axon interactions (Wang and Marquardt, 2013). For instance, the pre-target segregation of olfactory (Imai et al., 2009) or corpus callosal axons (Zhou et al., 2013) appears to be based on the patterned axonal expression of guidance receptors and their respective ligands and the sorting of dorsal root sensory axons relies on contact-dependent guidance with motor axons (Wang et al., 2011).

A large-scale genetic screen in zebrafish isolated several mutants that display defects in retinotectal topographic mapping (Baier et al., 1996; Karlstrom et al., 1996; Trowe et al., 1996). Among them was the *nevermind* (*nev*) mutant, which showed pathfinding errors of dorsal axons into the ventral tract and aberrant innervation of the lateral tectum leading to mapping defects in the tectum (Trowe et al., 1996). Subsequently, Pittman et al. (2010) showed that *nev* encodes cytoplasmic FMR1 interacting protein 2 (CYFIP2) and that the associated phenotype was cell autonomous. CYFIP2 is a member of a highly conserved gene family that has been genetically linked to autism spectrum disorder (ASD) and the Fragile X syndrome (FXS), the most common form of inherited intellectual disability (Abekhoukh and Bardoni, 2014). Human CYFIP2 is 98% identical to zebrafish CYFIP2, and zebrafish CYFIP1 is 86% identical to CYFIP2 (Pittman et al., 2010; Schenck et al., 2001). Analyses of *cyfip1*$^{+/-}$ and *cyfip2*$^{+/-}$ mice revealed a haploinsufficiency in both cases, resulting in Fragile-X-like abnormal dendritic spines (Bozdagi et al., 2012; Han et al., 2015). Indeed, CYFIP proteins were first identified as direct binding partners of the mRNA-binding protein FMRP (Fragile X mental retardation protein) (Schenck et al., 2001). Consistent with this interaction, CYFIP1 has been reported to regulate post-synaptic mRNA translation by acting as a non-canonical translation initiation factor 4E binding protein (4E-BP) and by repressing the expression of specific FMRP mRNA targets (De Rubeis et al., 2013; Napoli et al., 2008).

Interestingly, CYFIP proteins also appear to have a completely distinct mode of action: they act as canonical components of the WAVE regulatory complex (WRC), consisting of WAVE1/2/3 (WAS protein family member), NCKAP1, ABI1/2, and HSPC300 (also known as BRK1) (Takenawa and Suetsugu, 2007). Upon a conformational change induced by the small GTPase Rac1, it has been reported that CYFIP proteins trigger actin-related proteins 2 and 3 (Arp2/3)-dependent actin nucleation (Chen et al., 2010; Steffen et al., 2004). Recent evidence has

 © 2018 The Authors. Published by Elsevier Inc.
This is an open access article under the CC BY license (http://creativecommons.org/licenses/by/4.0/).

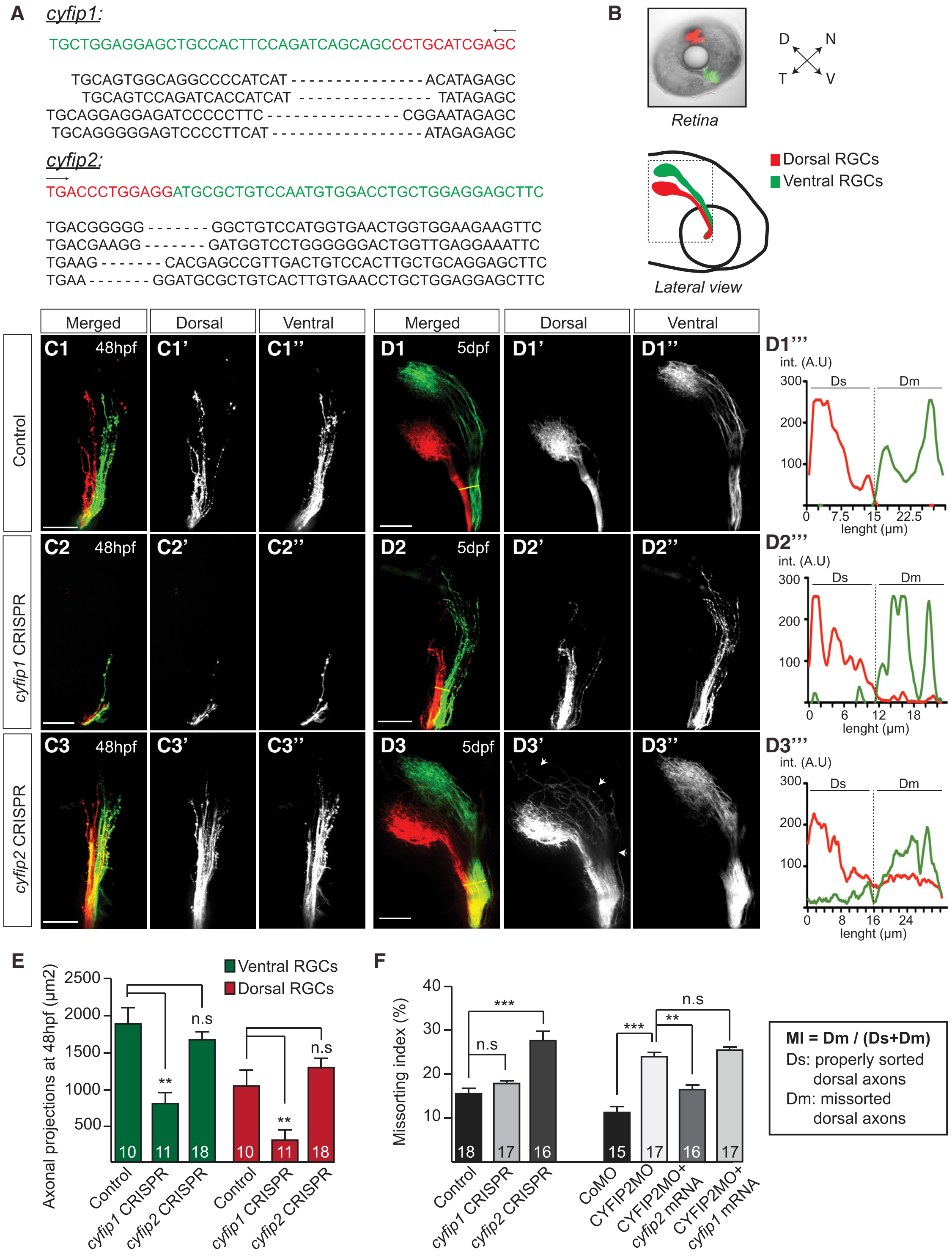

Figure 1. Differential Function of CYFIP1 and CYFIP2 during RGC Axonal Development

(A) Sequence analysis of the whole zebrafish embryo injected with *cas9* mRNA + *cyfip1* or *cyfip2* gRNAs. The target site is indicated on the sequence (green), followed by 4 examples of corresponding mutated regions.

(B) DiI (red) and DiO (green) fluorescent dyes were injected in the zebrafish embryo retina at 5 dpf. The dashed line denotes the confocal imaging area of the optic tract (OT).

(C1–D3‴) Dorsal (D) (C1′–C3′, D1′–D3′) and Ventral (V) (C1″–C3″, D1″–D3″) RGC projections were analyzed in control embryos (*cas9* mRNA + gRNA control) (C1, D1), *cyfip1* CRISPR-injected embryos (*cas9* mRNA + gRNA *cyfip1*) (C2, D2), and *cyfip2* CRISPR-injected embryos (*cas9* mRNA + gRNA *cyfip2*) (C3, D3) at 48 hpf

(legend continued on next page)

highlighted a role for CYFIP1 in regulating presynaptic activity during early stages of development (Hsiao et al., 2016), when both the WRC and Fragile-X-related RNA binding proteins are present (Antar et al., 2006; Njoo et al., 2015), and mutations in the *Drosophila* genes encoding dCYFIP, FMRP, and members of the WRC all give rise to similar axon guidance and synaptogenesis defects (Schenck et al., 2003, 2004). However, whether CYFIP2 possesses a similar dual role in coordinating mRNA translation and actin remodeling during neural wiring is not known.

Here we find that CYFIP1 and CYFIP2 act non-redundantly during RGC axon development, with CYFIP2 specifically involved in axon sorting. Using *in vivo* and *in vitro* approaches, we report that CYFIP2 regulates RGC axon segregation by coordinating homotypic fasciculation and heterotypic repulsive responses. CYFIP2 associates and is co-transported with ribonucleoprotein particles (RNPs) along the RGC axon shaft, and axon-axon contact promotes their entry into the growth cone. Once there, CYFIP2 translocates from RNPs to the WRC in the peripheral domain, where it regulates actin polymerization and filopodial dynamics. Rescue experiments with specific ablation of different CYFIP2 regulatory domains show that CYFIP2-mediated WRC activity, but not translational regulation, is necessary for pre-target axon sorting. Taken together, these data demonstrate how RGC axons integrate axon-axon contact sensing mechanisms through CYFIP2 to refine their relative positions in the optic tract.

RESULTS

Distinct Functions for CYFIP1 and CYFIP2 in RGC Axon Pathfinding *In Vivo*

To begin exploring the molecular mechanisms associated with optic tract (OT) axon sorting, we focused on CYFIP proteins. The *nev* (*cyfip2*) mutant exhibits dorso-ventral sorting errors in the OT and aberrant innervation of the lateral tectum (Pittman et al., 2010). CYFIP2 and CYFIP1 are very similar (86% identical in zebrafish), but it is not known whether CYFIP1 also functions in OT axon sorting. Comparison of the developmental expression patterns of the two CYFIP proteins with immunohistochemistry revealed distinct patterns (Figures S1A and S1B). In the retinal ganglion cell (RGC) layer, visualized by the expression of a membrane-targeted GFP (mGFP) under the control of atoh7 promoter (atoh7:gap-GFP), CYFIP1 expression peaks at 48 hr post-fertilization (hpf) and decreases to negligible levels by 72 hpf and 5 days post-fertilization (dpf) (Figure S1A). By contrast, CYFIP2 expression is detected at 48 hpf and increases to higher levels at 72 hpf and 5 dpf (Figure S1B), raising the possibility that the two proteins function differentially.

To test this idea, we used a CRISPR/Cas9 approach for targeted deletion of *cyfip1* and *cyfip2* genes (Shah et al., 2015). We selected specific guide RNAs (gRNAs) that mutated the second exon of the *cyfip1* or *cyfip2* locus by assessing their efficacy through sequencing of the targeted cutting sites in F_0 embryos. Both injections of *cyfip1* gRNA and *cyfip2* gRNA induce specific deletions of various sizes within the targeted sequences in respectively 50% and 70% of the embryos, validating the method (Figure 1A). Topographic sorting of RGC axons in the OT can be visualized in fixed whole-mount zebrafish larval brains by injection of lipophilic dyes DiI (red) and DiO (green) in the dorsal (D) and ventral (V) quadrants of the retina (Figure 1B) (Baier et al., 1996; Pittman et al., 2010). After contralateral eye removal, the corresponding axonal projections in the OT can then be analyzed in lateral view (Figure 1B). At 48 hpf, D and V axons begin to segregate in the OT (Figure 1C1), and by 5 dpf, this segregation is complete (Figure 1D1) (Poulain and Chien, 2013; Stuermer, 1988). In gRNA *cyfip1* + *cas9* mRNA-injected embryos, we observed a delay in axonal growth in 54.5% of embryos (n = 11 embryos) (Figure 1C2), resulting in a significant reduction of both D and V projections in the OT (Figure 1E). At 5 dpf, the same defect is observed in 56% of the embryos (n = 25 embryos), with fewer axons present in the OT and some axons failing to reach the tectum (Figure 1D2). Unlike the *nev* mutation, the absence of CYFIP1 does not affect D-V axonal sorting (Figure 1D2). In contrast, animals injected with the gRNA *cyfip2* show no effect on axonal extension at 48 hpf (n = 18 embryos) (Figures 1C3 and 1E) and 5 dpf (n = 16 embryos) (Figure 1D3), yet they exhibit D-V axonal sorting defects similar to the *nev* (*cyfip2*) mutant. Dorsal axons exhibit aberrant pathfinding at 48 hpf in the absence of CYFIP2 (Figure 1C3), leading to a large number of D axons missorting into the dorsal branch of the OT at 5 dpf (Figure 1D3). We measured the fluorescence signal intensity of D axons across the anterior-posterior axis of the OT (Figures 1D1‴, 1D2‴, and 1D3‴) to generate a missorting index (MI) (Poulain and Chien, 2013). Injection with gRNA *cyfip2*, but not gRNA *cyfip1*, induces a significant increase of the MI compared to control (Figure 1F).

To test for functional redundancy between the two CYFIP proteins *in vivo*, we performed a CYFIP2 knockdown and attempted to rescue the missorting phenotype by injection of mRNA for CYFIP1 or CYFIP2. Knockdown of CYFIP2 (60%–80%), achieved by injection of a splice-blocking morpholino (MO) antisense oligonucleotide to *cyfip2* (Figures S1C and S1E), phenocopies the gRNA *cyfip2* + *cas9* mRNA pre-target axon sorting defect without affecting CYFIP1 levels (Figures 1F and S1E). Quantification of the MI shows that co-injection of the CYFIP2MO with *cyfip2* mRNA, but not *cyfip1* mRNA, significantly rescues the axon missorting defect (Figure 1F). Together,

(C) and 5 dpf (D). Arrows in (D3′) show missorted dorsal axons in the OT. Yellow lines in (D1)–(D3) indicate the reference line used for quantification of the missorting index (MI). Examples of DiI (dorsal) and DiO signals plotted along the reference line corresponding to sorted (D1‴, D2‴) or misprojected (D3‴) RGC axons (int., intensity; A.U, Arbitrary Units).

(E) Quantifications of D and V axonal projection area in the OT at 48 hpf.

(F) The missorting index (MI) was quantified as the ratio of the intensity signal of the missorted D (Dm) axons to all the D axons (Dm+Ds). For gRNA *cyfip1*-injected embryos, only the embryos showing an axon growth phenotype were quantified (n = 18 embryos).

Error bars represent SEM. **p < 0.01, ***p < 0.001, n.s., non significant (Mann-Whitney test for E and F). The number of zebrafish analyzed is indicated on the bars. Scale bars: 50 μm (C1–D3). See also Figure S1.

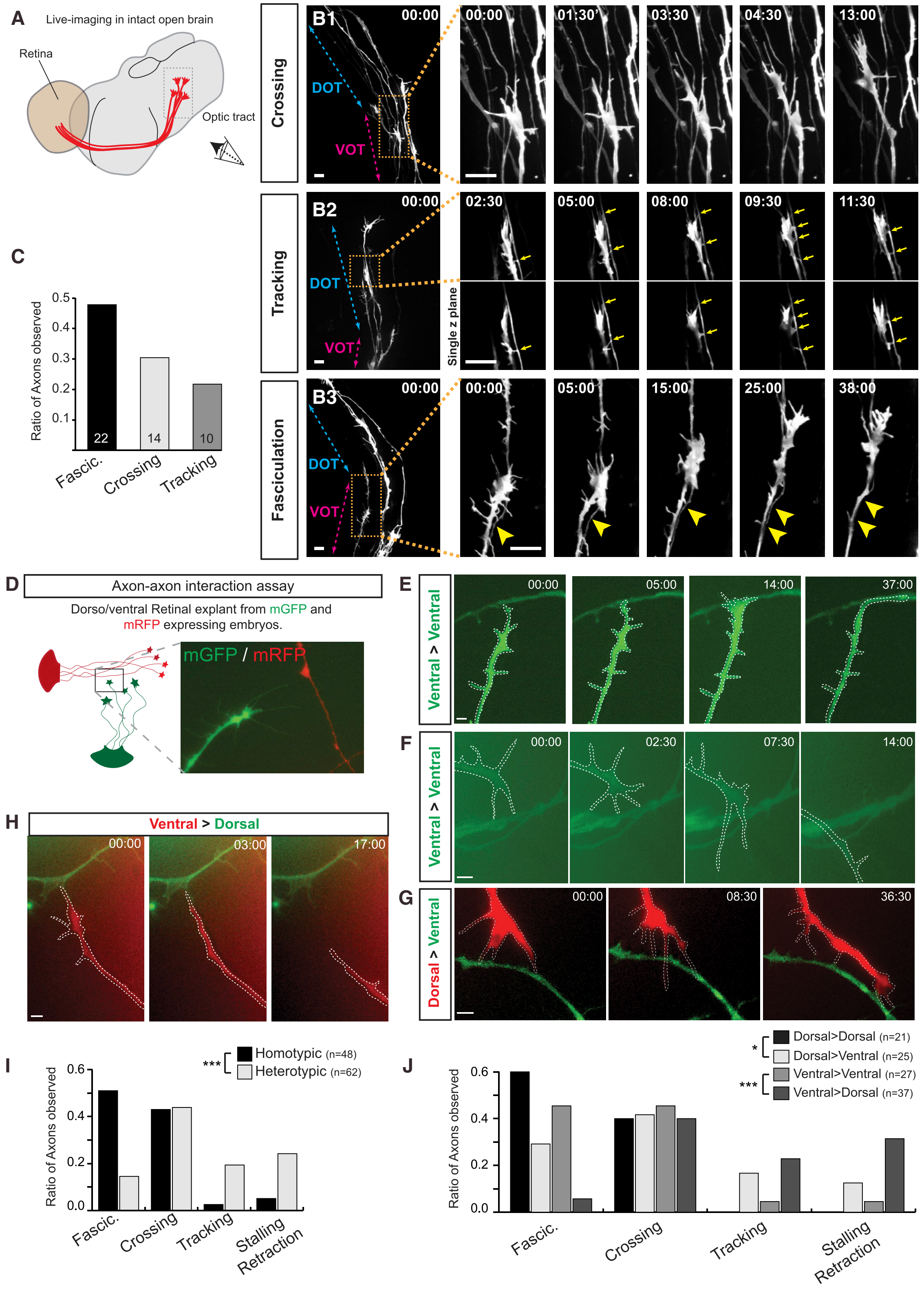

(legend on next page)

these results show differential expression and non-redundant function for CYFIP1 and CYFIP2 proteins during RGC axon development, with CYFIP2 specifically involved in axon sorting and CYFIP1 involved in axon extension.

Dorsal and Ventral RGCs Exhibit Homotypic and Heterotypic Axon-Axon Contact Recognition

CYFIP2 acts autonomously in pre-target axon sorting (Pittman et al., 2010) and since interactions between different retinal axons can trigger specific "recognition" responses *in vitro* (Bonhoeffer and Huf, 1980; Raper and Grunewald, 1990), we postulated that direct interactions between retinal axons facilitate, and possibly drive, topographic tract sorting. To address this, we performed time-lapse imaging to visualize the dynamic interactions between axonal growth cones and other retinal axons in the OT *in vivo*, using the intact (exposed) brain preparation of *Xenopus* (Figure 2A) (Chien et al., 1993; Wong et al., 2017). Indeed, topographic dorso-ventral RGC axon sorting also occurs in *Xenopus* (Fawcett et al., 1984) and owing to their bigger growth cone size compare to zebrafish, around twice the diameter, this method allowed us to visualize detailed growth cone behaviors *in vivo*. Eye-targeted electroporation (Falk et al., 2007) of mGFP was performed at stage 28, followed by time-lapse imaging in the OT 24 hr later (Figure 2A). Images of GFP-labeled growth cones were captured before, during, and after encounters with other GFP-labeled axons and a systematic analysis of their dynamic responses was performed (Figure 2B; Movie S1). Three distinct types of growth cone responses were identified following contact: (1) "crossing" events, during which the growth cone smoothly crosses over the contacted axon shaft, resulting in an antero-posterior displacement in the OT (Figure 2B1); (2) "tip-toe-tracking" events, characterized by the extension of multiple filopodia that alternately contact and withdraw from the axon shaft in contact, causing the growth cone to "tip-toe" along the axon while maintaining a distance from it (Figure 2B2); and (3) "axonal fasciculation" events, characterized by the growth cone adhering to and merging fully with the contacted axon, leading to a shared trajectory (Figure 2B3). Quantification revealed axonal fasciculation to be the most prevalent growth cone-axon encounter response occurring in the OT (47.83%), compared to crossing (30.43%) or tracking (21.74%) events (Figure 2C). These results show that growth cones exhibit a range of different behaviors following contact with other axons in the OT *in vivo*.

We next asked whether these different types of behavior are related to the topographic origin of the RGCs in the retina. For this, we took advantage of the ease of culturing *Xenopus* tissue and co-cultured retinal explants from different topographic regions of the retina (Figure 2D). Dorsal (D) and ventral (V) retinal explants from embryos expressing mRFP or mGFP were co-cultured, enabling D and V axons to be distinguished by color. Images of growth cones approaching and making first contact with axons were then captured with time-lapse microscopy. Quantification was performed using phase optics to avoid phototoxicity (Movie S2). We first compared homotypic (D->D and V->V) and heterotypic (D->V and V->D) axon interactions and found that homotypic contacts induce mostly fasciculation (Figure 2E) or crossing events (Figure 2F) with a similar ratio (0.51 and 0.43, respectively) (Figures 2I). In contrast, heterotypic interactions principally stall axonal growth, followed by tip-toe-tracking (Figure 2G) or retraction (Figures 2H and 2I). These results indicate that topographic origin underlies growth cone behavioral heterogeneity. The similarity of the responses *in vivo* and *in vitro* (fasciculation, tracking, and crossing) also indicates that they are not simply a peculiarity of the *in vitro* conditions. We next investigated whether there is any specificity to the type of contact responses exhibited by dorsal versus ventral growth cones. Analysis revealed that both populations exhibit homotypic and heterotypic axon-axon contact recognition (Figure 2J). Taken together, these results reveal that RGC axons exhibit a range of contact-induced responses related to their topographic origin.

CYFIP2 Regulates Both Homotypic Fasciculation and Heterotypic Repulsion

We next asked whether perturbing CYFIP2 levels could affect the different axon-axon interaction responses. We first verified that CYFIP2 protein is expressed in the *Xenopus* retina in a similar developmental pattern as zebrafish (Figures 3A and S1D). CYFIP2 knockdown (KD) was targeted to the central nervous system (CNS) by injecting CYFIP2MO into the dorsal blastomeres at the 4-cell stage and gave rise to a 50% decrease in the protein level without affecting CYFIP1 levels (Figure 3B). CYFIP2 KD was found to affect both homotypic and heterotypic axon-axon interactions (Figure 3C; Movie S3). In the homotypic condition, there is a significant reduction in fasciculation events and an increase in the number of axon stalling events (Figures 3C and 3D). Indeed, around 45% of RGC axons fail to adhere and

Figure 2. Dorsal and Ventral RGCs Exhibit Homo- and Heterotypic Axon-Axon Contact Recognition

(A) Schematic of the retinotectal projection in *Xenopus* embryo. Time-lapse imaging of the OT was done from the indicated lateral view.

(B1–B3) An example of each observed *in vivo* axon-axon response is showed first as an initial acquired large field, followed by a time-lapse sequence at high magnification. The position of the ventral (VOT) and dorsal (DOT) optic tract is indicated. (B1) RGC growth cone (GC) crossing the encounter axon shaft. (B2) RGC GC tracking along the encounter axon shaft by multiple filopodia contacts (yellow arrows). (B3) RGC GC growing on the encounter axon, leading to the fasciculation of the two axon shafts (yellow arrowheads).

(C) Quantification of the axon-axon responses observed in the optic tract.

(D) Assay used to monitor axon-axon interactions *in vitro*.

(E and F) Examples of fasciculation (E) and crossing (F) events observed during homotypic interactions.

(G and H) Examples of tracking (G) and retraction (H) events observed during heterotypic interactions.

(I) Quantification of the global homotypic and heterotypic responses.

(J) Quantification of the axon-axon responses relative to the RGC topographic origin. Time stamps are in the format of min:s. *$p < 0.05$, ***$p < 0.001$ (chi-square test for I and J). Numbers of events analyzed are indicated on the graph (n = 21 independent experiments).

Scale bars: 10 μm (B) and 5 μm (E–H).

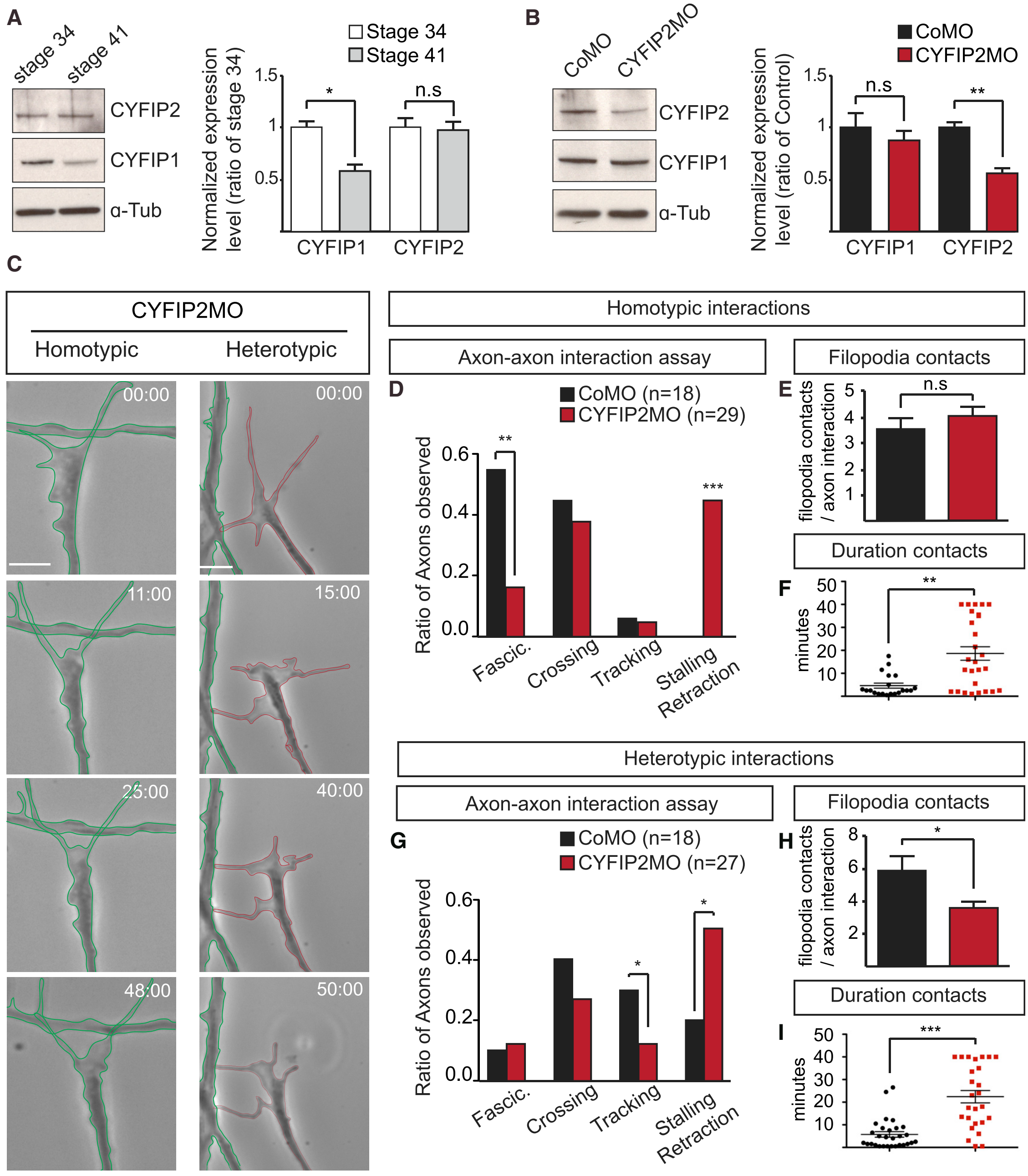

Figure 3. RGC Axon-Axon Interactions Require CYFIP2 Function

(A) Representative western blots and quantification of CYFIP1 and CYFIP2 levels in *Xenopus* eye lysates at stages 34 and 41 (n = 3, normalized to α-Tubulin).

(B) Representative western blots and quantification of CYFIP2 (n = 6, normalized to α-Tubulin) and CYFIP1 (n = 3, normalized to α-Tubulin) levels in CYFIP2MO-compared to CoMO-injected embryos at stage 34.

(C) Examples of stalling growth cones (GCs) during homotypic and heterotypic responses after CYFIP2 depletion.

(D) Quantification of the homotypic interaction responses after CYFIP2 knockdown.

(E and F) Quantification of the number (E) and duration (F) of filopodia contacts during fasciculation and stalling events in CYFIP2MO (n = 7 GC, n = 27 filopodia) compared to CoMO (n = 6 GC, n = 20 filopodia) conditions for homotypic interactions.

(G) Quantification of the heterotypic interaction responses after CYFIP2 knockdown.

(H and I) Quantification of the number (H) and duration (I) of filopodia contacts during tracking and stalling events in CYFIP2MO (n = 8 GC, n = 26 filopodia) compared to CoMO (n = 5 GC, n = 29 filopodia) conditions for heterotypic interactions.

(D and G) Numbers of events analyzed are indicated on the graph (n = 12 independent experiments).

(E–I) Error bars represent SEM. $^*p < 0.05$, $^{**}p < 0.01$, $^{***}p < 0.001$, n.s., non-significant (Mann-Whitney test for A, B, E, F, H and I) and (Fisher's exact test for D and G). Time stamps are in the format of min:s. Scale bars: 5 μm (C).

merge with the encountered axon shaft and stop their growth in response to cell contact (Figure 3C). Interestingly, we noticed that CYFIP2MO-associated axonal stalling is correlated with a significant increase in the duration of filopodial contacts (19.22 ± 3.02 min, n = 27 filopodia) compared to control growth cones exhibiting fasciculation (4.63 ± 1.10 min, n = 20 filopodia) (p = 0.0017, Mann-Whitney test) (Figure 3F). Analysis of the heterotypic responses shows a significant reduction of the tracking events and an increase in the proportion of stalling axons (Figures 3C and 3G). We found that this defect is associated with a decrease in filopodial contacts with the contacted axon (Figure 3C) compared to tracking growth cones in control conditions (Figure 2G; Movie S2). Indeed, quantification reveals a significant reduction of the number of filopodial contacts made by CYFIP2-depleted growth cones (3.6 ± 0.3 contacts) compared to control growth cones (5.8 ± 0.8 contacts) (Figure 3H). Moreover, the duration of the contacts is significantly increased in CYFIP2MO (22.6 ± 2.8 min, n = 26 filopodia) compared to CoMO condition (5.7 ± 1.2 min, n = 29 filopodia) (Figure 3I). These results indicate that CYFIP2 is important for normal growth cone responses during D-V axon sorting.

CYFIP2 Translocates to Growth Cone Periphery on Axon Contact and Regulates Actin Polymerization and Filopodial Dynamics

In isolated (non-contacted) growth cones, CYFIP2 is mainly restricted to the central domain with a weaker signal in the periphery and at the tips of filopodia and lamellipodia (Figures 4A). This signal is reduced by CYFIP2MO injection (Figures S2A and S2B). To examine the dynamics of CYFIP2 distribution in growth cones, we expressed a GFP-tagged fusion protein (CYFIP2-GFP) in RGCs and performed time-lapse imaging (Movie S4). Results show dynamic movements of the protein with transient accumulations occurring at the leading edge of lamellipodia where new filopodia arise (Figure 4B). By using a lipophilic dye to label the entire plasma membrane, we confirmed that CYFIP2-GFP accumulates at the tips of the majority of growth cone filopodia (75.62% ± 4.9%, n = 53 filopodia, n = 12 growth cones) (Figure 4C). In particular, CYFIP2-GFP is enriched at the tips of filopodia during extension (91.67% ± 4.4%, n = 53 filopodia, n = 12 growth cones) and then disappears or disperses before filopodial retraction (Figure 4C). We then asked whether its localization is regulated in response to axon-axon contact. Immunocytochemistry revealed a marked increase in CYFIP2 signal (approximately 75%) in growth cones that make contact with another axon shaft (Figure 4D). Moreover, the subcellular distribution of the protein changes after axon-axon contact, with a significant increase (~25%) in signal intensity in the periphery of the growth cone (Figures 4E–4G).

If CYFIP2 plays a direct role in filopodial dynamics, this could help explain its role in attractive and repulsive growth cone responses (Dent et al., 2011; McConnell et al., 2016). In line with this possibility, we found a significant decrease in the frequency of filopodial formation and retraction with CYFIP2 knockdown (Figure S2E). Moreover, in the absence of CYFIP2, there is an increase in the average filopodial lifetime (Figures S2H and S2I). These results suggest that CYFIP2 regulates the dynamic behavior of filopodia. When normal growth cones contact an axon shaft, they increase the number and length of filopodia (Figures 4H–4J). These contact-induced increases are abolished in absence of CYFIP2 (Figures 4H–4J). These results are in accordance with the defect in the number and duration of filopodial contacts during D-V axon sorting observed in CYFIP2-depleted axons (Figures 3F, 3H, and 3I). As growth cone filopodia are highly enriched in dynamic actin filaments (F-actin) (Dent et al., 2011), we measured the amount of polymerized actin in control and CYFIP2-depleted growth cones by phalloidin staining. We found an increase of F-actin in growth cones that encounter an axon shaft (Figures 4H and 4K). CYFIP2-depletion abolishes this increase in F-actin signal in response to axon-axon contact (Figures 4H and 4K), suggesting a prominent role for CYFIP2 in regulating actin polymerization during axon-axon interactions. Together, these results point to CYFIP2 as a modulator of growth cone periphery actin polymerization and filopodial dynamics triggered by axon-axon contact.

CYFIP2 Interacts with RNPs and the WRC in Distinct Subcellular Compartments

Biochemical and FRET experiments have shown that CYFIP1 associates with the WRC complex and RNPs to coordinate cytoskeletal remodeling and mRNA translation in dendrites (De Rubeis et al., 2013; Napoli et al., 2008). CYFIP2 also interacts with both the core components of the WRC (NAPK1, WAVE1, ABI2, and HSPC300) in HEK293 cells (Kumar et al., 2013) and RNA granules in the developing brain identified by proteomic analysis (Elvira et al., 2006). To distinguish which complex CYFIP2 associates with in RGC axons, we first searched for CYFIP2 molecular partners in *Xenopus* brain using a pull-down assay. We expressed CYFIP2-GFP by cDNA injection in dorsal blastomeres at 4-cell stage, and brains were collected from stage 35/36 embryos, corresponding to the stage when axons are growing in the OT. By GFP pull-down and western blot analysis, we detected the protein NCKAP1 in the co-precipitated material, confirming CYFIP2's presence in the WRC (Figure 5A). We also detected a positive signal for known constituents of RNPs, such as the *Xenopus* RNA binding protein Fragile X mental retardation-related protein (xFXR), ELAV-like proteins, and the ribosomal proteins Rps3A and Rpl10a (Figure 5A).

To map CYFIP2's interactions with these different complexes in axons, we performed co-immunostaining of CYFIP2 with NCKAP1 (WRC marker) or xFXR (RNP marker). In the RGC axon shaft, CYFIP2 shows a strong colocalization with xFXR and only a weak colocalization with NCKAP1 (Figures 5B and 5C). In the growth cone, CYFIP2 colocalizes with xFXR in the central domain (Figure 5D) but, in contrast, colocalizes with NCKAP1 in the peripheral domain (Figure 5E). These results suggest that CYFIP2 associates primarily with RNPs along the axon and in the central domain of the growth cone but switches to join the WRC in the growth cone periphery.

To confirm this, we performed proximity ligation assays (PLAs) on RGC axons, which enable detection of protein-protein interactions in fixed cells (Fredriksson et al., 2002; Yoon et al., 2012). In the axon shaft, PLA reveal a strong CYFIP2-xFXR signal compared to a sparse CYFIP2-NCKAP1 signal (Figures 5F and 5H). In the growth cone, CYFIP2 interacts with both NCKAP1 and xFXR (Figures 5G and 5I) but the CYFIP2-NCKAP1-PLA signal is prominent in the peripheral domain whereas the

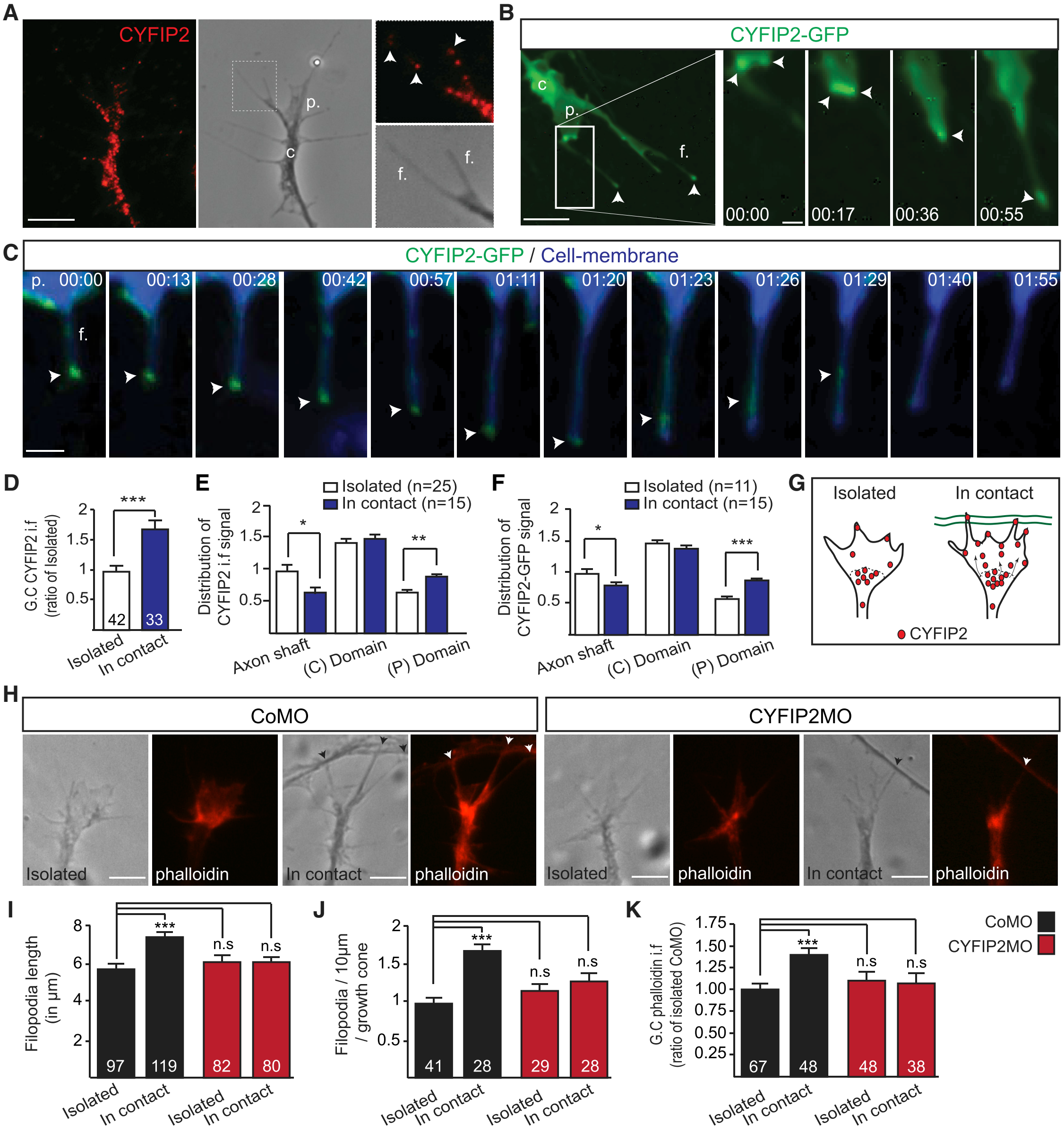

Figure 4. CYFIP2 Regulates Filopodial Dynamics and F-actin in the Growth Cone Peripheral Domain upon Axon-Axon Contact

(A) CYFIP2 immunostaining on stage 32 *Xenopus* retinal growth cone (GC). Arrowheads indicate CYFIP2 in filopodia.

(B) Time-lapse imaging of CYFIP2-GFP movements in RGC GC. Arrowheads indicate the accumulation of CYFIP2-GFP.

(C) Time-lapse imaging of CYFIP2-GFP movements in an elongating GC filopodia labeled by a membrane marker (blue). Arrowheads indicate CYFIP2-GFP accumulation.

(D) Quantification of CYFIP2 signal intensity in GC.

(E) Distribution of CYFIP2 signal intensity along RGC axon shaft (last 10 µm) and GC central (C) and peripheral (P) domains.

(F) Distribution of CYFIP2-GFP signal intensity along RGC axon shaft (last 10 µm) and GC central (C) and peripheral (P) domains.

(G) Scheme illustrating the observed relocalization of CYFIP2 in the GC peripheral domain during axon-contact.

(H) Phalloidin immunostaining on isolated or in contact stage 32 *Xenopus* retinal GCs from CoMO- and CYFIP2MO-injected embryos.

(I and J) Quantifications of filopodia length (I) and number (J).

(K) Quantification of phalloidin signal intensity in GC.

The numbers of GC (D, J, and K) or filopodia (I) analyzed are indicated on the bars. Error bars represent SEM. *$p < 0.05$, **$p < 0.01$, ***$p < 0.001$, n.s., non-significant (Mann-Whitney test for D–F and I–K). The GC central domain (c.), peripheral domain (p.), and filopodia (f.) are indicated (A–C). Time stamps are in the format of min:s. Scale bars: 5 µm (A, B left panel, and H); 1 µm (B right panels); 2 µm (H). See also Figure S2.

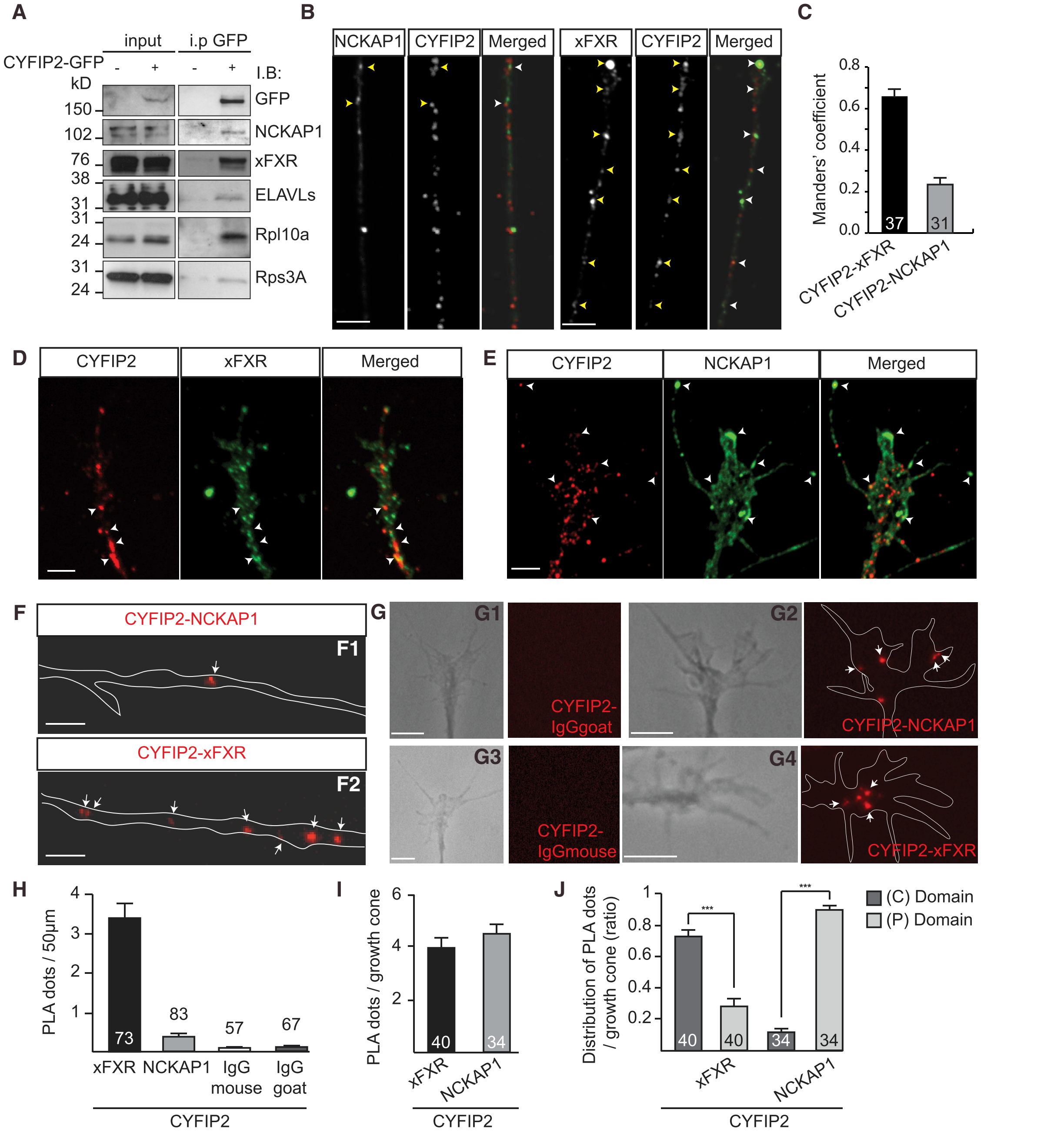

Figure 5. Subcellular Interactions of CYFIP2 with RNPs and WRC in RGC Axons

(A) Immunoprecipitation with anti-GFP was performed on stage 32 *Xenopus* brains expressing CYFIP2-GFP, and immunoblotted with the indicated antibodies.
(B) CYFIP2 and NCKAP1 immunostainings on stage 32 *Xenopus* retinal cultures.
(C) Protein colocalization quantified by Manders' coefficient.
(D and E) CYFIP2 with xFXR (D) or NCKAP1 (E) immunostainings on RGC growth cones.
(F) Representative examples of the proximity ligation assay (PLA) obtained between CYFIP2 and NCKAP1, or CYFIP2 and xFXR, in the axon shaft.
(G) Representative PLA examples obtained between CYFIP2 and NCKAP1 (G2) or control IgGgoat (G1), and between CYFIP2 and xFXR (G4) or control IgGmouse (G3) in the GC.
(H) Quantification of the PLA signals in the axon shaft.
(I) Quantification of the PLA signals in the GC.
(J) Ratios of the PLA signals in the central (C) and peripheral (P) domain of the GC (Mann-Whitney test, ***$p < 0.001$).
Error bars represent SEM. Number of axons analyzed is indicated on the graph. Scale bars: 5 µm.

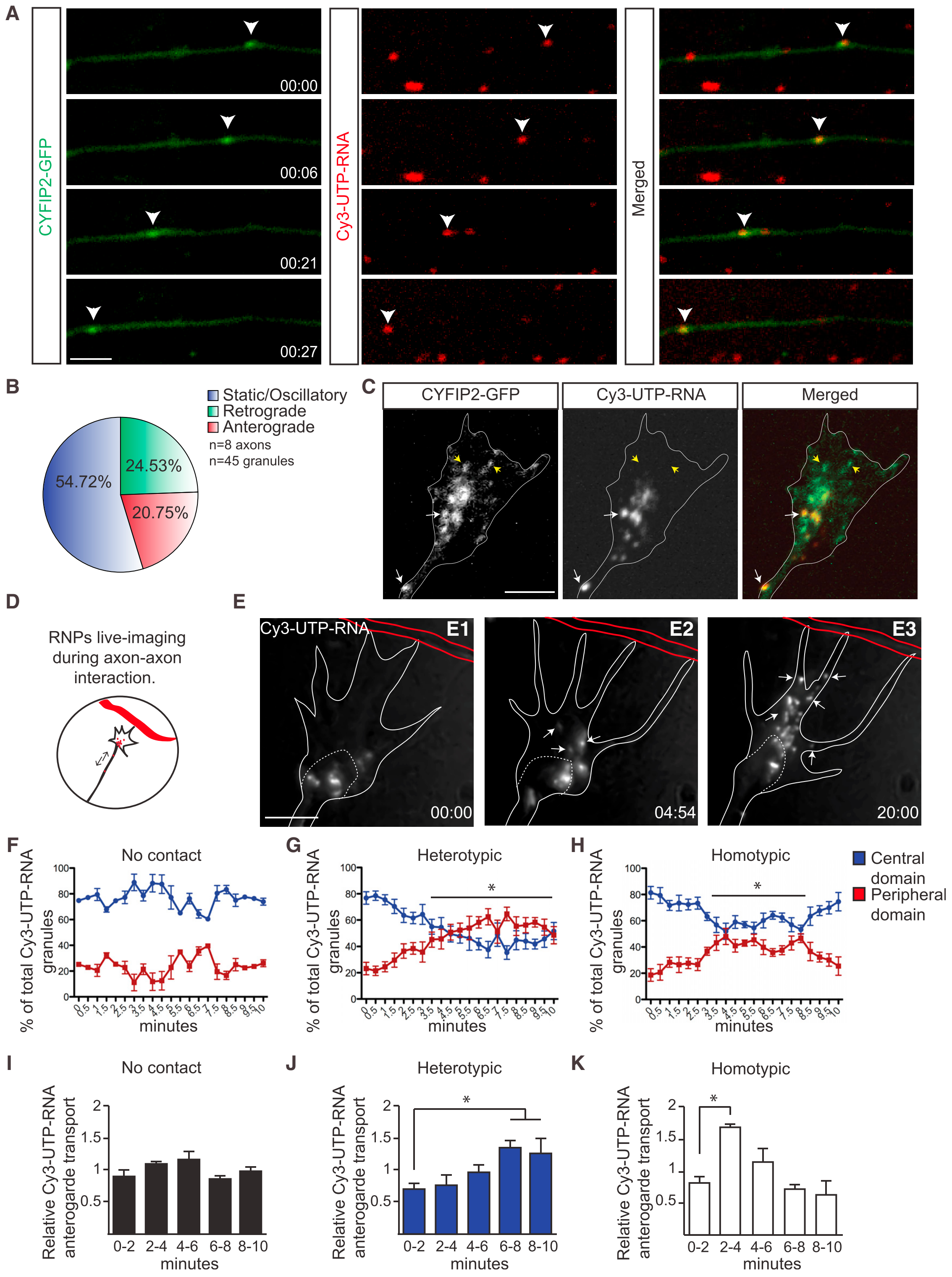

Figure 6. CYFIP2-RNP Recruitment in Growth Cones on Axon-Axon Contact

(A) Time-lapse sequence showing the transport of CYFIP2-GFP in Cy3-UTP-RNA-granules in RGC axon shaft, highlighted by arrowheads.

(B) Quantification of the CYFIP2-GFP containing RNPs motions in the RGC axon shaft.

(C) Example of CYFIP2-GFP and Cy3-UTP-RNA granules signals in a RGC growth cone (GC).

(D) Assay used to follow Cy3-UTP-RNA granules movements during axon-axon interactions.

(legend continued on next page)

CYFIP2-xFXR-PLA signal is strongest in the central domain (Figure 5J). Thus, CYFIP2 appears to interact with different partners in distinct subcellular compartments.

CYFIP2 Co-traffics with RNA and Axon-Axon Contact Alters Transport Dynamics

The increase of CYFIP2 levels in growth cones in response to axon-axon contact and its preferential association with xFXR (RNPs) in the axon shaft suggest that RNPs themselves respond to axon-axon interactions. Indeed, previous studies have shown that regulated RNA transport can be crucial in facilitating some cue-mediated axonal guidance responses (Leung et al., 2006; Welshhans and Bassell, 2011; Willis et al., 2007). We, therefore, asked whether CYFIP2 associates with RNAs in living axons. To do so, we labeled endogenous RNA by injection of uridine-5′-triphosphate (UTP) tagged with a fluorescent marker (Cy3-UTP) (Piper et al., 2015; Wong et al., 2017) (Figure 6A). In retinal explants from embryos co-injected with CYFIP2-GFP plasmid and Cy3-UTP, we found that 84% of CYFIP2-GFP puncta are associated with RNA granules in the axon shaft (Figure 6A). Moreover, time-lapse imaging shows that CYFIP2-GFP and Cy3-UTP-labeled RNPs are actively co-transported (Figure 6A; Movie S5). Just over half of CYFIP2-containing Cy3-UTP-RNA granules exhibit static/oscillatory displacements (54.72%), while the rest show motile trafficking along the axon in both retrograde (24.53%) and anterograde (20.75%) directions (Figure 6B). Once inside the growth cone, CYFIP2-GFP signals are strongly associated with Cy3-UTP-RNA granules in the central domain but not in the peripheral domain (Figure 6C).

These results suggest that CYFIP2 is transported along RGC axons by coupling to RNPs and prompted us to quantify the relative distribution of the Cy3-UTP-RNA granules in the growth cones (Figure 6D; Movie S6). In isolated growth cones without cell contacts, the majority of Cy3-UTP-RNA granules localize to the central domain (76.4% ± 1.6%), with few in the peripheral domain (23.6% ± 1.6%) (Figure 6F), consistent with the spatial distribution of mRNA transcripts in growth cones (Zivraj et al., 2010). Upon heterotypic axon-axon contact, the relative distribution of Cy3-UTP-RNA granules changes substantially. At 3.5 min after contact, a significant increase in the relative amount of Cy3-UTP-RNA granules is observed in the peripheral domain (45.15% ± 5.67%) (Figures 6E2 and 6G), compared to the time of initial contact (23.19% ± 4.75%) (Figures 6E1 and 6G). The RNA granules then progressively invade the periphery of the growth cone, maintaining a significant change in distribution over 10 min (Figure 6G), which persists to later time points (Figure 6E3). During homotypic interactions, we also found a significant increase in the relative percentage of granules in the periphery of the growth cone from 3 min post-contact (36.75% ± 3.03%), compared to that at the time of the initial contact (18.64% ± 4.8%) (Figure 6H). However, in contrast with heterotypic contacts, this redistribution is transient and no significant difference is observed after 8 min (Figure 6H). We then asked whether the contact-induced effect on growth cone RNA dynamics also correlates with a change in axonal transport. Quantification of the number of RNA granules reaching the growth cone shows a constant anterograde transport in the absence of axon contact (Figure 6I). However, during heterotypic axon contact there is a progressive increase in anterograde transport of Cy3-UTP-RNA granules (Figure 6J), with a significant effect 6 min after axon-contact. Intriguingly, homotypic interactions result in different RNA dynamics, with a transient increase after 2 min that progressively returns to baseline after 6 min (Figure 6K). These results reveal a specific recruitment of RNPs toward the growth cone in response to axon-axon contact, like CYFIP2. The co-trafficking and parallel contact-induced behaviors suggest that CYFIP2 is shuttled around on RNA granules in axons.

CYFIP2 Regulation of the WAVE Complex Is Critical for RGC Pre-target Axon Sorting

Since CYFIP2 can associate with RNPs and the WRC, it seems possible that either or both local translation and actin remodeling are involved in CYFIP2's role in topographic axon sorting. To discriminate between these two pathways, we used mutated forms of CYFIP2 to uncouple the two regulatory functions of the protein (Figure 7A). Insertion of a Lys727Glu point mutation (CYFIP2-mutE) is sufficient to reduce the interaction with eIF4E, but not with the WRC, thus inhibiting CYFIP2's ability to regulate translation (De Rubeis et al., 2013; Hsiao et al., 2016; Napoli et al., 2008) (Figures 7A and S3). Disruption of CYFIP2's regulation of actin dynamics is achieved by removing the C-terminal part of the protein (CYFIP2-ΔCTD), which partially reduces CYFIP2's regulation of mRNA translation but totally abolishes its interaction with the WRC (De Rubeis et al., 2013; Hsiao et al., 2016; Napoli et al., 2008) (Figures 7A and S3).

We verified the effects of these mutations on CYFIP2 localization and trafficking in RGC retinal explants (Figure 7B). Like endogenous CYFIP2, CYFIP2-GFP is present in axons, enriched in the growth cone central domain and sparse in the peripheral domain (Figure 7B; Movie S4). CYFIP2-GFP-ΔCTD is similarly present in axons and enriched in the growth cone central domain. However, it fails to accumulate in the peripheral domain of the growth cone, consistent with a requirement for the C-terminal domain in mediating the WRC interaction and CYFIP2's subsequent peripheral localization (Figure 7B). In contrast, CYFIP2-GFP-mutE shows a similar localization to CYFIP2-GFP-WT, even in the growth cone periphery, consistent with its retained ability to interact with both RNPs and WRC, despite the inhibition of its translational control (Figures 7B and S3).

To determine which of CYFIP2's two distinct functions underlies pre-target axon sorting *in vivo*, we performed knockdown of CYFIP2 with a rescue using wild-type CYFIP2 or the two different

(E) Example of a time-lapse sequence showing the recruitment of Cy3-UTP-RNA granules in the peripheral domain of the GC in response to a heterotypic contact.
(F–H) Quantifications of the relative Cy3-UTP-RNA granules distribution for each time point in isolated GCs (F) (n = 3 experiments), after heterotypic interactions (G) (n = 5 experiments) or homotypic interactions (H) (n = 5 experiments). T = 0 correspond to the cell-contact point.
(I–K) Quantification of Cy3-UTP-RNA granules anterograde transport over time in isolated (I) (n = 3 experiments), after heterotypic interactions (J) (n = 4 experiments) or homotypic interactions (K) (n = 4 experiments), normalized to the average anterograde transport for each axon.
Error bars represent SEM. *p < 0.05 (Mann-Whitney test for F–K). Time stamps are in the format of min:s. Scale bars: 3 μm (A) and 5 μm (C and E).

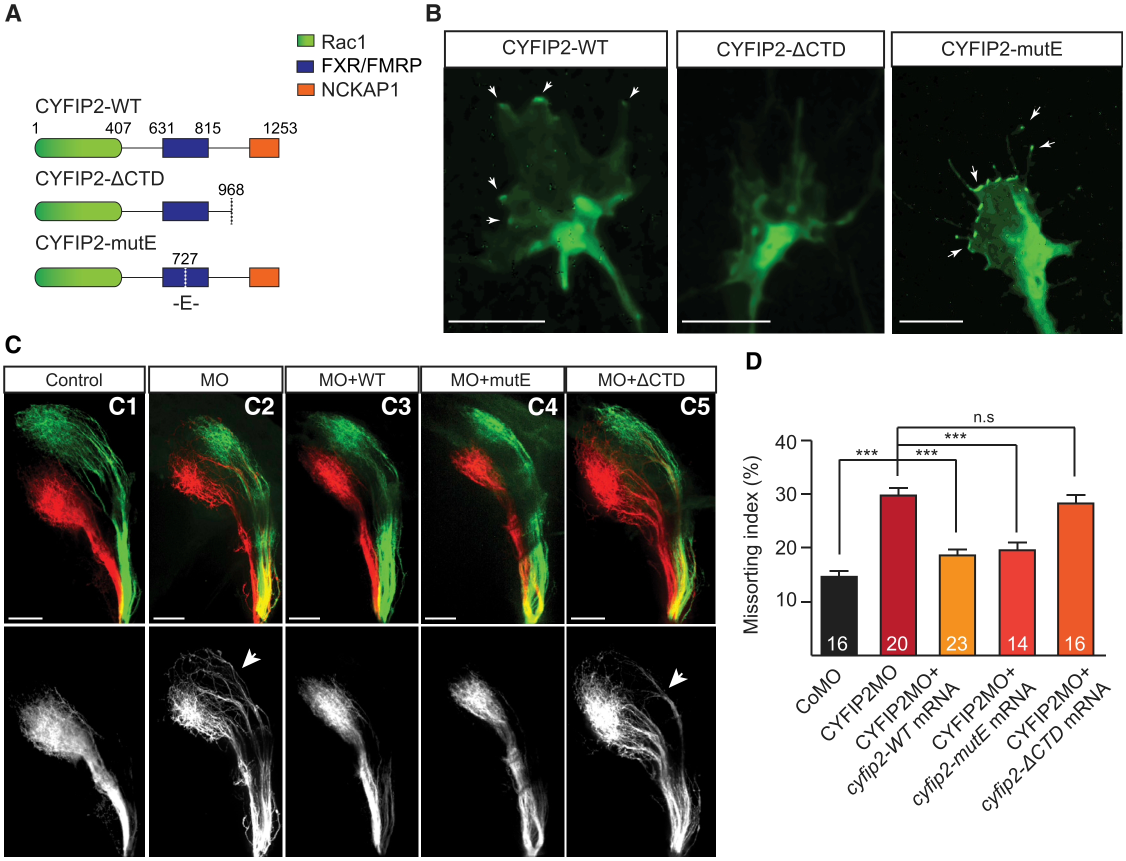

Figure 7. CYFIP2 Regulation of the WRC Mediates Axon Sorting in the Tract

(A) Schematic illustrating CYFIP2's regulatory domains and mutations.

(B) Representative examples of CYFIP2WT-GFP (n = 9 GC), CYFIP2ΔCTD-GFP (n = 11 GC), and CYFIP2mutE-GFP (n = 13 GC) expression in *Xenopus* retinal cultures (n = 4 experiments). Arrows indicate CYFIP2-GFP accumulation in the growth cone peripheral domain and filopodia.

(C1–C5) Lateral-view of whole-mount 5 dpf zebrafish embryos injected with DiI and DiO in the dorsal and ventral retina, respectively. Co-injection of CYFIP2MO + *CYFIP2WT* (C3) or *CYFIP2mutE* (C4), but not *CYFIP2ΔCTD* (C5), mRNAs rescue the pre-sorting defect observed in CYFIP2MO-injected embryos (C2) compared to CoMO-injected embryos (C1).

(D) Quantification of the Missorting Index (Mann-Whitney test, ***$p < 0.001$, n.s., non-significant).

Error bars represent SEM. The number of zebrafish analyzed is indicated on the bars. Scale bars: 5 μm (B) or 50 μm (C). See also Figures S3 and S4.

CYFIP2 mutants in zebrafish and analyzed the D-V axon topography. Embryos were co-injected with CYFIP2MO and *cyfip2-wt*, *cyfip2-ΔCTD*, or *cyfip2-mutE* mRNAs and OT axon sorting was quantified using the missorting index (Figures 7C and 7D). As previously found, *cyfip2-wt* is able to rescue axon missorting (Figure 7C3). The same result is obtained by co-injecting *cyfip2-mutE* mRNA (Figure 7C4). The *cyfip2-ΔCTD* mRNA, on the other hand, is not able to rescue the missorting phenotype (Figure 7C5). This suggests that CYFIP2-mediated actin remodeling, but not translational regulation, is required for D-V axon sorting. In line with these results, both genetic deletion (*ztor*) and pharmacological inhibition of TOR complex 1 activity, a master regulator of axonal mRNA translation (Shigeoka et al., 2013), does not result in D-V axon missorting (Figures S4A and S4B). Filopodial dynamics are also unaffected by acute protein synthesis inhibition (Figures S4C and S4D), indicating that CYFIP2-associated functions described in this system are mostly insensitive to intra-axonal regulation of translation. In contrast, filopodial dynamics are almost completely abolished when actin polymerization is inhibited by low concentration of cytochalasin D, which is consistent with a primary role of actin remodeling in this process (Figure S4D) (McConnell et al., 2016). Taken together, the results demonstrate that CYFIP2 controls RGC tract axon sorting through its regulation of the WRC.

DISCUSSION

Axon pre-target sorting is a common feature of CNS axon tracts that facilitates topographic matching between pre-synaptic neurons and their post-synaptic targets. In this study, we show that retinal axon sorting in the optic tract involves axon-axon fasciculation typical of homotypic interactions and repulsion typical of heterotypic interactions. CYFIP2, but not its homolog CYFIP1, is necessary for these differential responses and for axon sorting in the optic tract. When growth cones encounter other axons, CYFIP2 moves into the growth cone peripheral domain where

it associates with the WRC and regulates actin and filopodial dynamics. Finally, we show that it is this association that is essential for its role in axon sorting.

The first *in vitro* analysis of RGC axon-axon interactions reported that temporal axons were repelled by a repulsive cue present on the surface of nasal axons (Bonhoeffer and Huf, 1980; Raper and Grunewald, 1990). This behavior was subsequently found to be driven by axonal ephrinAs/EphAs signaling and important for map formation in the superior colliculus (Suetterlin and Drescher, 2014), a mechanism that appears distinct from tract sorting (Pittman et al., 2010; Plas et al., 2008). In this study, we focused on dorsal and ventral axons as, unlike nasal and temporal axons, they are clearly segregated along the anterior to posterior axis of the optic tract before reaching the target zone. Our *in vitro* and *in vivo* results show that axon-axon fasciculation and repulsive tracking coordinately regulate this process. Interestingly, our results suggest that CYFIP2 affects both types of responses by regulating filopodial dynamics. Fine-tuning of growth cone filopodia extension and retraction is essential for proper attractive and repulsive responses to extrinsic cues *in vitro*, and guidance factors are known to affect filopodial dynamics locally (Dent et al., 2011; McConnell et al., 2016). However, retinal growth cones deficient in Ena/Vasp function have few or no filopodia yet can still navigate correctly to the optic tectum *in vivo* (Dwivedy et al., 2007), indicating that long-range pathfinding does not require normal filopodial dynamics. In accordance with these results, no obvious defect of long-distance axonal growth and guidance is observed in the absence of CYFIP2. However, there are errors in topographic sorting within the optic tract. This suggests that CYFIP2 regulation of growth cone filopodial dynamics is involved in the process of axon-axon sorting within the optic tract.

Growth cone filopodial dynamics directly depend on the regulation of actin filament assembly and turnover in the peripheral domain, where almost all the features of actin organizational changes rely on actin binding proteins (ABPs) (Gomez and Letourneau, 2014; Mattila and Lappalainen, 2008). In RGCs, we found that CYFIP2 interacts with the WRC in the peripheral domain of the growth cone. The activation of the WRC relies on Rac1 binding to CYFIP proteins, allowing the release of WAVE1 from the complex. WAVE1 triggers Arp2/3-dependent actin nucleation, which generates branched actin networks in lamellipodia (Korobova and Svitkina, 2008). Growth cone filopodia formation is also dependent on WAVE proteins (Goh et al., 2012; Nozumi et al., 2003) and Arp2/3 is localized at the bases of filopodia in the growth cone, where it regulates filopodia formation (Korobova and Svitkina, 2008; San Miguel-Ruiz and Letourneau, 2014). Interestingly, our results show an accumulation of CYFIP2 at the tips of elongating filopodia. This is consistent with the localization of the WAVE proteins in the growth cone (Nozumi et al., 2003) but, puzzlingly, Arp2/3 function would not be expected to be required in filopodia where F-actin is unbranched and raises the possibility of an Arp2/3-independent actin regulatory function of the WRC at filopodial tips (Korobova and Svitkina, 2008; Sasaki et al., 2000). CYFIP2 also regulates the synthesis of multiple proteins, yet inhibition of intra-axonal protein synthesis does not seem to affect filopodial dynamics in the growth cone in agreement with Spillane et al. (Spillane et al., 2012). These results combined with the findings that CYFIP2 function in axon sorting depends on its ability to regulate the WRC-dependent actin dynamics, and not translation, strengthens the idea that CYFIP2's regulation of filopodia is key in this process.

Axon sorting has been observed in diverse nerve tracts, including the olfactory nerve (Satoda et al., 1995), the corpus callosum (Zhou et al., 2013), and the thalamo-cortical tract (Lokmane et al., 2013). In these cases, axon segregation has been found to result from asymmetrical expression of guidance receptors and their respective ligands, expressed intrinsically in the axons of different neuron subpopulations. In the olfactory system, Neuropilin-1 and its repulsive ligand, Sema-3A, are expressed on different sets of axons, which help them sort into an olfactory map within the tract (Imai et al., 2009). Neuropilin-1 is also expressed in retinal axons and recent evidence suggests that it may also be involved in topographic sorting within the optic tract (Hörnberg et al., 2016). It is therefore possible that CYFIP2 works downstream of Neuropilin-1 signaling. Indeed, a consensus motif WRC-interacting receptor surface (WIRS) present on a large class of receptors has been identified, allowing the direct recruitment of the fully assembled WRC to receptors (Chen et al., 2014). However, the WIRS motif does not appear to be present in Neuropilin-1. There are more than a hundred WIRS motif-containing transmembrane proteins, many of which are expressed in RGCs, opening up the possibility that direct binding between CYFIP2-containing WRC and receptors underlies retinotectal pre-target axon sorting.

In the mouse brain, CYFIP1 and CYFIP2 have similar expression patterns including expression in the outer layers of the cortex, hippocampus, striatum, and cerebellum and both proteins are localized at dendritic spines and enriched at excitatory synapses (Han et al., 2015; Pathania et al., 2014). Haploinsufficiency of *cyfip1* affects cortical and hippocampal dendritic spine maturation and is associated with an enhanced metabotropic glutamate receptor (mGluR)-dependent long-term depression (Bozdagi et al., 2012; De Rubeis et al., 2013). Interestingly, analysis of *cyfip2*+/− mice revealed a defect of cortical spine morphogenesis, but not hippocampal spines, suggesting cell-type-specific roles of the two CYFIP proteins (Han et al., 2015). In this study, we found a differential regulation of CYFIP1 and CYFIP2 expression during retina development that appears to be conserved in mice (Shigeoka et al., 2016), suggesting potential non-redundant functions of the two proteins. Our results show that genetic deletion of *cyfip1* and *cyfip2* in zebrafish results in mutually exclusive defects in RGC axonal growth and topographic sorting, respectively, implying that CYFIP1 and CYFIP2 are responsible for different, and potentially complementary, functions in the retinotectal system. Expression of CYFIP1 also fails to rescue the pretarget axon sorting phenotype associated with CYFIP2 depletion, suggesting that the two proteins have different interactomes and may be regulated differently. For example, unlike CYFIP1, CYFIP2 binds to the two FMRP paralogs FXR1p and FXR2p (Schenck et al., 2001). The FXR proteins diverge in their RNA-binding protein properties, with regulatory differences that appear to be dependent on the target mRNA, developmental stage, and cellular subtype (Guo et al., 2015; Guo et al., 2011; Menon and Mihailescu, 2007). It is possible, therefore, that CYFIP proteins exert independent functions within different RNPs.

Intriguingly, another possibility relevant to our study is the presence of specific CYFIP-containing WRC complexes. Indeed, specific WRC isoforms can be assembled from the combination of different paralogs of each component (Takenawa and Suetsugu, 2007). The WAVE isoforms WAVE1, WAVE2, and WAVE3 have been found to exhibit differential localization in neuronal growth cones (Nozumi et al., 2003). WAVE1 is distributed uniformly throughout lamellipodia, whereas WAVE2 and WAVE3 are concentrated at the tips of protruding filopodia, as we report here for CYFIP2. Thus, CYFIP-containing WRC complexes might differ in their composition and interacting regulators, allowing precise spatial control of their activity in the growth cone. Future analyses of the CYFIP1 and CYFIP2 interactomes at the cellular and subcellular (axons, dendrites, and soma) levels will be needed to better understand their differential roles.

In this study, we found that CYFIP2 associates with RNPs in RGC axons, is co-transported with RNPs, and is recruited to the growth cone in response to axon-axon contacts. CYFIP2 has homology with the translation repressors, the eIF4E binding proteins (eIF4E-BPs) (Napoli et al., 2008), and can potentially help to silence mRNA translation as the RNP is trafficked along the axon. Interestingly, like CYFIP, Mena has recently been shown to have a dual function in regulating both actin polymerization and mRNA translation (Vidaki et al., 2017). Mena, but not its paralogs VASP and EVL, associates with a specific set of mRNAs and is a key regulator of Dyrk1a synthesis in axons in response to BDNF stimulation (Vidaki et al., 2017). It is intriguing to speculate that actin-regulatory proteins may coordinately control actin and translation in response to cues. However, our results indicate that CYFIP2's regulation of the WRC, but not local translation, is required for RGC tract axonal sorting. This is consistent with the absence of axon extension or sorting defects observed in the *ztor* mutant. Recent evidence showed that acute pharmacological inhibition of protein synthesis does not affect growth cone navigation in the optic tract but impairs terminal axonal branching in the tectum, which may demand higher levels of locally synthesized proteins (Wong et al., 2017). It is therefore possible that despite not being required for axon sorting, CYFIP2-dependent coordination of actin remodeling and translation regulation could play a role during later phases of neural wiring. These data also suggest an intriguing model in which CYFIP2 protein "hitchhikes" with RNPs for trafficking in growing RGC axons in order to reach the growth cone, where the protein performs translation-independent functions. It has been shown that various stimuli, such as synaptic activity and external cues, can induce rapid RNP transport to specific sites in axons and dendrites. Our results suggest that CYFIP2 proteins can exploit this highly regulated trafficking process to increase its levels in a specific subcellular domain and, once "on-site," perform an essential role in optic tract sorting by regulating axon-axon responses.

STAR★METHODS

Detailed methods are provided in the online version of this paper and include the following:

- KEY RESOURCES TABLE
- CONTACT FOR REAGENT AND RESOURCE SHARING
- EXPERIMENTAL MODEL AND SUBJECT DETAILS
  - Zebrafish embryo maintenance
  - *Xenopus laevis* embryo maintenance
- METHOD DETAILS
  - DNA constructs, generation of mRNAs and morpholinos
  - gRNAs design and quantification of CRISPR efficiency
  - *Xenopus* and zebrafish embryo injection
  - Lipophilic dye labeling and imaging
  - Electroporation and *in vivo* analysis of axon-axon interactions
  - *Xenopus* Retinal explant cultures
  - Live imaging in *Xenopus* retinal ganglion cell axons
  - Pharmacological treatments
  - Immunostainings and proximity ligation assay
  - Immunoprecipitation and puromycin assay
- QUANTIFICATIONS AND STATISTICAL ANALYSIS
  - Statistics
  - *In vivo* axonal projection and Missorting index
  - *In vivo* analysis of axon-axon interactions
  - Axon-axon interaction assay
  - CYFIP2-GFP and RNA dynamics
  - Analysis of filopodia dynamics
  - Immunocytochemistry and PLA analysis

SUPPLEMENTAL INFORMATION

Supplemental Information includes four figures and six movies and can be found with this article online at https://doi.org/10.1016/j.neuron.2018.01.027.

ACKNOWLEDGMENTS

We thank Dr. Khandjian for the FXR antibody and Katrin Mooslehner for assistance with molecular cloning. This work was supported by Wellcome Trust Programme Grants (085314, C.E.H.), Wellcome Trust Investigator Award (SIA 100329/Z/12/Z, W.A.H.), European Research Council Advanced Grant (322817, C.E.H.), EMBO Long Term Fellowship (ALTF849-2013, J.-M.C.), Cambridge Trust, Croucher Foundation, and Sir Edward Youde Memorial Fund (H.H.W.).

AUTHOR CONTRIBUTIONS

Conceptualization, J.-M.C., W.H., and C.E.H.; Methodology, J.-M.C., D.B., H.H.W., and C.E.H.; Formal analysis, J.-M.C. and D.B.; Investigation, J.-M.C., L.K., H.H.W., and D.B.; Resources, J.-M.C., H.H.W., D.B., W.H., and C.E.H; Writing-Original Draft, J.-M.C., W.H., and C.E.H; Supervision, J.-M.C., W.H., and C.E.H; Funding Acquisition, W.H. and C.E.H.

DECLARATION OF INTERESTS

The authors declare no conflict of interest.

Received: March 6, 2017
Revised: November 10, 2017
Accepted: January 11, 2018
Published: March 7, 2018

REFERENCES

Abekhoukh, S., and Bardoni, B. (2014). CYFIP family proteins between autism and intellectual disability: links with Fragile X syndrome. Front. Cell. Neurosci. *8*, 81.

Antar, L.N., Li, C., Zhang, H., Carroll, R.C., and Bassell, G.J. (2006). Local functions for FMRP in axon growth cone motility and activity-dependent regulation of filopodia and spine synapses. Mol. Cell. Neurosci. *32*, 37–48.

Baier, H., Klostermann, S., Trowe, T., Karlstrom, R.O., Nüsslein-Volhard, C., and Bonhoeffer, F. (1996). Genetic dissection of the retinotectal projection. Development *123*, 415–425.

Bonhoeffer, F., and Huf, J. (1980). Recognition of cell types by axonal growth cones in vitro. Nature *288*, 162–164.

Boulanger, J., Kervrann, C., Bouthemy, P., Elbau, P., Sibarita, J.B., and Salamero, J. (2010). Patch-based nonlocal functional for denoising fluorescence microscopy image sequences. IEEE Trans. Med. Imaging *29*, 442–454.

Bozdagi, O., Sakurai, T., Dorr, N., Pilorge, M., Takahashi, N., and Buxbaum, J.D. (2012). Haploinsufficiency of Cyfip1 produces fragile X-like phenotypes in mice. PLoS ONE *7*, e42422.

Chen, Z., Borek, D., Padrick, S.B., Gomez, T.S., Metlagel, Z., Ismail, A.M., Umetani, J., Billadeau, D.D., Otwinowski, Z., and Rosen, M.K. (2010). Structure and control of the actin regulatory WAVE complex. Nature *468*, 533–538.

Chen, B., Brinkmann, K., Chen, Z., Pak, C.W., Liao, Y., Shi, S., Henry, L., Grishin, N.V., Bogdan, S., and Rosen, M.K. (2014). The WAVE regulatory complex links diverse receptors to the actin cytoskeleton. Cell *156*, 195–207.

Chien, C.B., Rosenthal, D.E., Harris, W.A., and Holt, C.E. (1993). Navigational errors made by growth cones without filopodia in the embryonic Xenopus brain. Neuron *11*, 237–251.

Das, T., Payer, B., Cayouette, M., and Harris, W.A. (2003). In vivo time-lapse imaging of cell divisions during neurogenesis in the developing zebrafish retina. Neuron *37*, 597–609.

De Rubeis, S., Pasciuto, E., Li, K.W., Fernández, E., Di Marino, D., Buzzi, A., Ostroff, L.E., Klann, E., Zwartkruis, F.J., Komiyama, N.H., et al. (2013). CYFIP1 coordinates mRNA translation and cytoskeleton remodeling to ensure proper dendritic spine formation. Neuron *79*, 1169–1182.

Dent, E.W., Gupton, S.L., and Gertler, F.B. (2011). The growth cone cytoskeleton in axon outgrowth and guidance. Cold Spring Harb. Perspect. Biol. *3*, 3.

Ding, Y., Sun, X., Huang, W., Hoage, T., Redfield, M., Kushwaha, S., Sivasubbu, S., Lin, X., Ekker, S., and Xu, X. (2011). Haploinsufficiency of target of rapamycin attenuates cardiomyopathies in adult zebrafish. Circ. Res. *109*, 658–669.

Dwivedy, A., Gertler, F.B., Miller, J., Holt, C.E., and Lebrand, C. (2007). Ena/VASP function in retinal axons is required for terminal arborization but not pathway navigation. Development *134*, 2137–2146.

Elvira, G., Wasiak, S., Blandford, V., Tong, X.K., Serrano, A., Fan, X., del Rayo Sánchez-Carbente, M., Servant, F., Bell, A.W., Boismenu, D., et al. (2006). Characterization of an RNA granule from developing brain. Mol. Cell. Proteomics *5*, 635–651.

Falk, J., Drinjakovic, J., Leung, K.M., Dwivedy, A., Regan, A.G., Piper, M., and Holt, C.E. (2007). Electroporation of cDNA/Morpholinos to targeted areas of embryonic CNS in Xenopus. BMC Dev. Biol. *7*, 107.

Fawcett, J.W., Taylor, J.S., Gaze, R.M., Grant, P., and Hirst, E. (1984). Fibre order in the normal Xenopus optic tract, near the chiasma. J. Embryol. Exp. Morphol. *83*, 1–14.

Feldheim, D.A., and O'Leary, D.D. (2010). Visual map development: bidirectional signaling, bifunctional guidance molecules, and competition. Cold Spring Harb. Perspect. Biol. *2*, a001768.

Fredriksson, S., Gullberg, M., Jarvius, J., Olsson, C., Pietras, K., Gústafsdóttir, S.M., Ostman, A., and Landegren, U. (2002). Protein detection using proximity-dependent DNA ligation assays. Nat. Biotechnol. *20*, 473–477.

Goh, W.I., Lim, K.B., Sudhaharan, T., Sem, K.P., Bu, W., Chou, A.M., and Ahmed, S. (2012). mDia1 and WAVE2 proteins interact directly with IRSp53 in filopodia and are involved in filopodium formation. J. Biol. Chem. *287*, 4702–4714.

Gomez, T.M., and Letourneau, P.C. (2014). Actin dynamics in growth cone motility and navigation. J. Neurochem. *129*, 221–234.

Guo, W., Zhang, L., Christopher, D.M., Teng, Z.Q., Fausett, S.R., Liu, C., George, O.L., Klingensmith, J., Jin, P., and Zhao, X. (2011). RNA-binding protein FXR2 regulates adult hippocampal neurogenesis by reducing Noggin expression. Neuron *70*, 924–938.

Guo, W., Polich, E.D., Su, J., Gao, Y., Christopher, D.M., Allan, A.M., Wang, M., Wang, F., Wang, G., and Zhao, X. (2015). Fragile X proteins FMRP and FXR2P control synaptic GluA1 expression and neuronal maturation via distinct mechanisms. Cell Rep. *11*, 1651–1666.

Han, K., Chen, H., Gennarino, V.A., Richman, R., Lu, H.C., and Zoghbi, H.Y. (2015). Fragile X-like behaviors and abnormal cortical dendritic spines in cytoplasmic FMR1-interacting protein 2-mutant mice. Hum. Mol. Genet. *24*, 1813–1823.

Hörnberg, H., Cioni, J.M., Harris, W.A., and Holt, C.E. (2016). Hermes regulates axon sorting in the optic tract by post-trancriptional regulation of Neuropilin 1. J. Neurosci. *36*, 12697–12706.

Hsiao, K., Harony-Nicolas, H., Buxbaum, J.D., Bozdagi-Gunal, O., and Benson, D.L. (2016). Cyfip1 Regulates Presynaptic Activity during Development. J. Neurosci. *36*, 1564–1576.

Huot, M.E., Bisson, N., Davidovic, L., Mazroui, R., Labelle, Y., Moss, T., and Khandjian, E.W. (2005). The RNA-binding protein fragile X-related 1 regulates somite formation in Xenopus laevis. Mol. Biol. Cell *16*, 4350–4361.

Imai, T., Yamazaki, T., Kobayakawa, R., Kobayakawa, K., Abe, T., Suzuki, M., and Sakano, H. (2009). Pre-target axon sorting establishes the neural map topography. Science *325*, 585–590.

Karlstrom, R.O., Trowe, T., Klostermann, S., Baier, H., Brand, M., Crawford, A.D., Grunewald, B., Haffter, P., Hoffmann, H., Meyer, S.U., et al. (1996). Zebrafish mutations affecting retinotectal axon pathfinding. Development *123*, 427–438.

Korobova, F., and Svitkina, T. (2008). Arp2/3 complex is important for filopodia formation, growth cone motility, and neuritogenesis in neuronal cells. Mol. Biol. Cell *19*, 1561–1574.

Kumar, V., Kim, K., Joseph, C., Kourrich, S., Yoo, S.H., Huang, H.C., Vitaterna, M.H., de Villena, F.P., Churchill, G., Bonci, A., and Takahashi, J.S. (2013). C57BL/6N mutation in cytoplasmic FMRP interacting protein 2 regulates cocaine response. Science *342*, 1508–1512.

Leung, K.M., van Horck, F.P., Lin, A.C., Allison, R., Standart, N., and Holt, C.E. (2006). Asymmetrical beta-actin mRNA translation in growth cones mediates attractive turning to netrin-1. Nat. Neurosci. *9*, 1247–1256.

Lokmane, L., Proville, R., Narboux-Nême, N., Györy, I., Keita, M., Mailhes, C., Léna, C., Gaspar, P., Grosschedl, R., and Garel, S. (2013). Sensory map transfer to the neocortex relies on pretarget ordering of thalamic axons. Curr. Biol. *23*, 810–816.

Mattila, P.K., and Lappalainen, P. (2008). Filopodia: molecular architecture and cellular functions. Nat. Rev. Mol. Cell Biol. *9*, 446–454.

McConnell, R.E., Edward van Veen, J., Vidaki, M., Kwiatkowski, A.V., Meyer, A.S., and Gertler, F.B. (2016). A requirement for filopodia extension toward Slit during Robo-mediated axon repulsion. J. Cell Biol. *213*, 261–274.

McLaughlin, T., Hindges, R., and O'Leary, D.D. (2003). Regulation of axial patterning of the retina and its topographic mapping in the brain. Curr. Opin. Neurobiol. *13*, 57–69.

Menon, L., and Mihailescu, M.R. (2007). Interactions of the G quartet forming semaphorin 3F RNA with the RGG box domain of the fragile X protein family. Nucleic Acids Res. *35*, 5379–5392.

Napoli, I., Mercaldo, V., Boyl, P.P., Eleuteri, B., Zalfa, F., De Rubeis, S., Di Marino, D., Mohr, E., Massimi, M., Falconi, M., et al. (2008). The fragile X syndrome protein represses activity-dependent translation through CYFIP1, a new 4E-BP. Cell *134*, 1042–1054.

Nieuwkoop, P.D., and Faber, J. (1994). Normal Table of Xenopus laevis (Daudin): A Systematical and Chronological Survey of the Development from the Fertilized Egg till the End of Metamorphosis (Garland Pub.).

Njoo, C., Agarwal, N., Lutz, B., and Kuner, R. (2015). The cannabinoid receptor CB1 interacts with the WAVE1 complex and plays a role in actin dynamics and structural plasticity in neurons. PLoS Biol. *13*, e1002286.

Nozumi, M., Nakagawa, H., Miki, H., Takenawa, T., and Miyamoto, S. (2003). Differential localization of WAVE isoforms in filopodia and lamellipodia of the neuronal growth cone. J. Cell Sci. *116*, 239–246.

Pathania, M., Davenport, E.C., Muir, J., Sheehan, D.F., López-Doménech, G., and Kittler, J.T. (2014). The autism and schizophrenia associated gene CYFIP1 is critical for the maintenance of dendritic complexity and the stabilization of mature spines. Transl. Psychiatry *4*, e374.

Piper, M., Lee, A.C., van Horck, F.P., McNeilly, H., Lu, T.B., Harris, W.A., and Holt, C.E. (2015). Differential requirement of F-actin and microtubule cytoskeleton in cue-induced local protein synthesis in axonal growth cones. Neural Dev. *10*, 3.

Pittman, A.J., Gaynes, J.A., and Chien, C.B. (2010). nev (cyfip2) is required for retinal lamination and axon guidance in the zebrafish retinotectal system. Dev. Biol. *344*, 784–794.

Plas, D.T., Lopez, J.E., and Crair, M.C. (2005). Pretarget sorting of retinocollicular axons in the mouse. J. Comp. Neurol. *491*, 305–319.

Plas, D.T., Dhande, O.S., Lopez, J.E., Murali, D., Thaller, C., Henkemeyer, M., Furuta, Y., Overbeek, P., and Crair, M.C. (2008). Bone morphogenetic proteins, eye patterning, and retinocollicular map formation in the mouse. J. Neurosci. *28*, 7057–7067.

Poggi, L., Vitorino, M., Masai, I., and Harris, W.A. (2005). Influences on neural lineage and mode of division in the zebrafish retina in vivo. J. Cell Biol. *171*, 991–999.

Poulain, F.E., and Chien, C.B. (2013). Proteoglycan-mediated axon degeneration corrects pretarget topographic sorting errors. Neuron *78*, 49–56.

Raper, J.A., and Grunewald, E.B. (1990). Temporal retinal growth cones collapse on contact with nasal retinal axons. Exp. Neurol. *109*, 70–74.

San Miguel-Ruiz, J.E., and Letourneau, P.C. (2014). The role of Arp2/3 in growth cone actin dynamics and guidance is substrate dependent. J. Neurosci. *34*, 5895–5908.

Sasaki, N., Miki, H., and Takenawa, T. (2000). Arp2/3 complex-independent actin regulatory function of WAVE. Biochem. Biophys. Res. Commun. *272*, 386–390.

Satoda, M., Takagi, S., Ohta, K., Hirata, T., and Fujisawa, H. (1995). Differential expression of two cell surface proteins, neuropilin and plexin, in Xenopus olfactory axon subclasses. J. Neurosci. *15*, 942–955.

Schenck, A., Bardoni, B., Moro, A., Bagni, C., and Mandel, J.L. (2001). A highly conserved protein family interacting with the fragile X mental retardation protein (FMRP) and displaying selective interactions with FMRP-related proteins FXR1P and FXR2P. Proc. Natl. Acad. Sci. USA *98*, 8844–8849.

Schenck, A., Bardoni, B., Langmann, C., Harden, N., Mandel, J.L., and Giangrande, A. (2003). CYFIP/Sra-1 controls neuronal connectivity in Drosophila and links the Rac1 GTPase pathway to the fragile X protein. Neuron *38*, 887–898.

Schenck, A., Qurashi, A., Carrera, P., Bardoni, B., Diebold, C., Schejter, E., Mandel, J.L., and Giangrande, A. (2004). WAVE/SCAR, a multifunctional complex coordinating different aspects of neuronal connectivity. Dev. Biol. *274*, 260–270.

Scholes, J.H. (1979). Nerve fibre topography in the retinal projection to the tectum. Nature *278*, 620–624.

Shah, A.N., Davey, C.F., Whitebirch, A.C., Miller, A.C., and Moens, C.B. (2015). Rapid reverse genetic screening using CRISPR in zebrafish. Nat. Methods *12*, 535–540.

Shigeoka, T., Lu, B., and Holt, C.E. (2013). Cell biology in neuroscience: RNA-based mechanisms underlying axon guidance. J. Cell Biol. *202*, 991–999.

Shigeoka, T., Jung, H., Jung, J., Turner-Bridger, B., Ohk, J., Lin, J.Q., Amieux, P.S., and Holt, C.E. (2016). Dynamic axonal translation in developing and mature visual circuits. Cell *166*, 181–192.

Sperry, R.W. (1963). Chemoaffinity in the orderly growth of nerve fiber patterns and connections. Proc. Natl. Acad. Sci. USA *50*, 703–710.

Spillane, M., Ketschek, A., Donnelly, C.J., Pacheco, A., Twiss, J.L., and Gallo, G. (2012). Nerve growth factor-induced formation of axonal filopodia and collateral branches involves the intra-axonal synthesis of regulators of the actin-nucleating Arp2/3 complex. J. Neurosci. *32*, 17671–17689.

Steffen, A., Rottner, K., Ehinger, J., Innocenti, M., Scita, G., Wehland, J., and Stradal, T.E. (2004). Sra-1 and Nap1 link Rac to actin assembly driving lamellipodia formation. EMBO J. *23*, 749–759.

Stuermer, C.A. (1988). Retinotopic organization of the developing retinotectal projection in the zebrafish embryo. J. Neurosci. *8*, 4513–4530.

Suetterlin, P., and Drescher, U. (2014). Target-independent ephrina/EphA-mediated axon-axon repulsion as a novel element in retinocollicular mapping. Neuron *84*, 740–752.

Takenawa, T., and Suetsugu, S. (2007). The WASP-WAVE protein network: connecting the membrane to the cytoskeleton. Nat. Rev. Mol. Cell Biol. *8*, 37–48.

Trowe, T., Klostermann, S., Baier, H., Granato, M., Crawford, A.D., Grunewald, B., Hoffmann, H., Karlstrom, R.O., Meyer, S.U., Müller, B., et al. (1996). Mutations disrupting the ordering and topographic mapping of axons in the retinotectal projection of the zebrafish, Danio rerio. Development *123*, 439–450.

Vidaki, M., Drees, F., Saxena, T., Lanslots, E., Taliaferro, M.J., Tatarakis, A., Burge, C.B., Wang, E.T., and Gertler, F.B. (2017). A requirement for mena, an actin regulator, in local mRNA translation in developing neurons. Neuron *95*, 608–622.e5.

Wang, L., and Marquardt, T. (2013). What axons tell each other: axon-axon signaling in nerve and circuit assembly. Curr. Opin. Neurobiol. *23*, 974–982.

Wang, L., Klein, R., Zheng, B., and Marquardt, T. (2011). Anatomical coupling of sensory and motor nerve trajectory via axon tracking. Neuron *71*, 263–277.

Welshhans, K., and Bassell, G.J. (2011). Netrin-1-induced local β-actin synthesis and growth cone guidance requires zipcode binding protein 1. J. Neurosci. *31*, 9800–9813.

Willis, D.E., van Niekerk, E.A., Sasaki, Y., Mesngon, M., Merianda, T.T., Williams, G.G., Kendall, M., Smith, D.S., Bassell, G.J., and Twiss, J.L. (2007). Extracellular stimuli specifically regulate localized levels of individual neuronal mRNAs. J. Cell Biol. *178*, 965–980.

Wong, H.H., Lin, J.Q., Ströhl, F., Roque, C.G., Cioni, J.M., Cagnetta, R., Turner-Bridger, B., Laine, R.F., Harris, W.A., Kaminski, C.F., and Holt, C.E. (2017). RNA docking and local translation regulate site-specific axon remodeling in vivo. Neuron *95*, 852–868.e8.

Yoon, B.C., Jung, H., Dwivedy, A., O'Hare, C.M., Zivraj, K.H., and Holt, C.E. (2012). Local translation of extranuclear lamin B promotes axon maintenance. Cell *148*, 752–764.

Zhou, J., Wen, Y., She, L., Sui, Y.N., Liu, L., Richards, L.J., and Poo, M.M. (2013). Axon position within the corpus callosum determines contralateral cortical projection. Proc. Natl. Acad. Sci. USA *110*, E2714–E2723.

Zivraj, K.H., Tung, Y.C., Piper, M., Gumy, L., Fawcett, J.W., Yeo, G.S., and Holt, C.E. (2010). Subcellular profiling reveals distinct and developmentally regulated repertoire of growth cone mRNAs. J. Neurosci. *30*, 15464–15478.

Zolessi, F.R., Poggi, L., Wilkinson, C.J., Chien, C.B., and Harris, W.A. (2006). Polarization and orientation of retinal ganglion cells in vivo. Neural Dev. *1*, 2.

STAR★METHODS

KEY RESOURCES TABLE

REAGENT or RESOURCE	SOURCE	IDENTIFIER
Antibodies		
anti-CYFIP2	Abcam	Cat# ab79716, RRID: AB_10673468
anti-CYFIP1	Abcam	Cat# ab108220, RRID: AB_10859239
anti-NCKAP1	Abcam	Cat# ab140856, RRID: AB_2721067
anti-xFXR	Huot et al., 2005	N/A
anti-ELAVLs	Santa Cruz Biotechnology	Cat# sc-5261, RRID: AB_627770
Anti-RPL10a	Proteintech Group	Cat# 16681-1-AP, RRID: AB_2181281
Anti-RPS3A	Proteintech Group	Cat# 14123-1-AP, RRID: AB_2253921
anti-puromycin	Millipore	Cat# MABE343, RRID: AB_2566826
anti-tubulin	Sigma-Aldrich	Cat# T6074, RRID: AB_477582
Anti-GFP	Abcam	Cat# ab6556, RRID: AB_305564
HRP-conjugated secondary antibody	Abcam	Cat# ab6789, RRID: AB_955439
HRP-conjugated secondary antibody	Abcam	Cat# ab97080, RRID: AB_10679808
Chemicals, Peptides, and Recombinant Proteins		
Cycloheximide	Sigma-Aldrich	Cat# C4859
Rapamycin	Calbiochem	Cat# 553210
Cytochalasin D	Sigma-Aldrich	Cat# C8273
Cy3-UTP	PerkinElmer	Cat# NEL582001EA
1,1’-Dioctadecyl-3,3,3′,3′-Tetramethylindocarbocyanine Perchlorate (DiI)	Thermo Fisher Scientific	Cat# D282
3,3′-dioctadecyloxacarbocyanine perchlorate (DiO)	Thermo Fisher Scientific	Cat# D275
Puromycin	Sigma-Aldrich	Cat# P8833
Dynabeads Protein G	Life Technologies	Cat# 10004D
Critical Commercial Assays		
mMESSAGE mMACHINE SP6 Transcription Kit	Thermo Fisher Scientific	Cat# AM1340
Gibson Assembly cloning kit	New England BioLabs	Cat# E5510S
QuikChange II Site-directed Mutagenesis Kit	Agilent Technologies	Cat# 200555
RNeasy Mini Kit	QIAGEN	Cat# 74104
MEGAscript T7 transcription Kit	Thermo Fisher Scientific	Cat# AM1334
RNA clean & concentrator-5	Zymo Research	Cat# R1016
Phire Animal Tissue Direct PCR Kit	Thermo Fisher Scientific	Cat# F140WH
DUOlink *in situ* PLA kit	Sigma-Aldrich	Cat# DUO92014
Experimental Models: Organisms/Strains		
Xenopus laevis	Nasco	https://www.enasco.com/p/LM00715MX/; https://www.enasco.com/p/LM00535MX/
Zebrafish: Tg(atoh7:gapGFP)	Zolessi et al., 2006	ZFIN: ZDB-ALT-070129-1
Zebrafish: *zTOR*	Ding et al., 2011	ZFIN: ZDB-ALT-120412-1
Oligonucleotides		
Morpholino: *Xenopus* CYFIP2 TTACCAAGTCCGGTAGCGACAGTCT	Gene Tools	N/A
Morpholino: Control CCTCTTACCTCAGTTACAATTTATA	Gene Tools	N/A
Morpholino: zebrafish CYFIP2 AGTGCATTAGGACGTGTACCTGGTA	Gene Tools; Pittman et al., 2010	N/A

(*Continued on next page*)

Continued

REAGENT or RESOURCE	SOURCE	IDENTIFIER
CYFIP1gRNA: GGAGGGCAGAGGCTCGATGC	Sigma-Aldrich	N/A
CYFIP2gRNA: GACAACCCACGTGACCCTGG	Sigma-Aldrich	N/A
Primer: sequencing zebrafish CYFIP1<br>5′ GCCATGTCTCACATGTGTTTTT 3′<br>5′ GCAGATACAGAAGAAGGGTTGC 3′	Sigma-Aldrich	N/A
Primer: sequencing zebrafish CYFIP2<br>5′ AGGTCATGACATTTCCCTTGTC 3′<br>5′ TCAGTGCATTAGGACGTGTACC 3′	Sigma-Aldrich	N/A
Recombinant DNA		
pCS2+mGFP	Das et al., 2003	N/A
pCS2+mRFP	Poggi et al., 2005	N/A
pCS2+CYFIP2-GFP	This paper	N/A
pCS2+CYFIP2-CTD-GFP	This paper	N/A
pCS2+CYFIP2mutE-GFP	This paper	N/A
pCS2-Cas9	Addgene	Cat# 47322
Xenopus CYFIP2	Source Bioscience	IRBHp990E0729D
CYFIP1	Dharmcon/GE Healthcare	MHS6278-202827458
CYFIP2	Source Bioscience	IRAUp969A0861D
Software and Algorithms		
Volocity	v.6.3.1	Volocity 3D Image Analysis Software, RRID: SCR_002668
Fiji	v.2.0.0-rc-43/1.51n	Fiji, RRID: SCR_002285
GraphPad PRISM	v.6.01	GraphPad Prism, RRID: SCR_002798
ND-SAFIR	Boulanger et al., 2010	http://serpico.rennes.inria.fr/doku.php?id=software:nd-safir:index

CONTACT FOR REAGENT AND RESOURCE SHARING

Further information and requests for resources and reagents should be directed to and will be fulfilled by the Lead Contact, Professor Christine E. Holt (ceh33@cam.ac.uk).

EXPERIMENTAL MODEL AND SUBJECT DETAILS

Zebrafish embryo maintenance

Zebrafish embryos of either sex were obtained from natural matings of Wild-type (AB-TL or TL), Tg(atoh7:gapGFP) or *zTOR (xu015)* (Ding et al., 2011) strains and raised at 28.5°C in E3 embryo medium. Embryos used for fluorescent imaging had the embryo medium supplemented with 0.003% phenylthiourea (PTU, Sigma) for pigment reduction. All animal work was approved by Local Ethical Review Committee at the University of Cambridge and performed according to the protocols of project license PPL 80/2198.

Xenopus laevis embryo maintenance

Xenopus laevis embryos were obtained by *in vitro* fertilization, raised in 0.1X Modified Barth's Saline (MBS) at 14°C-20°C and staged according to the tables of (Nieuwkoop and Faber, 1994). All animal work was approved by Local Ethical Review Committee at the University of Cambridge and performed according to the protocols of project license PPL 80/2198.

METHOD DETAILS

DNA constructs, generation of mRNAs and morpholinos

All constructs were expressed in the pCS2+ vector (David Turner, University of Michigan, Ann Arbor). We used the previously described pCS2+mGFP (Das et al., 2003) and pCS2+RFP (Poggi et al., 2005). *Xenopus cyfip2* cDNA (GenBank: AF107889) was obtained from addgene and sublconed in pCS2+ with an N-terminally Enhanced Green Fluorescent Protein (CYFIP2-GFP). The CYFIP2-CTD and CYFIP2mutE mutants were generated as previously described for CYFIP1 in (De Rubeis et al., 2013; Napoli et al., 2008). CYFIP2-CTD is a truncated mutated form of CYFIP2-GFP in which the C-terminal part of the protein is missing (968-1253). The

CYFIP2WT construct was digested with XmaI + XbaI to serve as a vector and the insert was PCR amplified (forward primer: 5′-CCAAGGCATGAATATGGCTCTCTGACGTGCACCAGATCTGCTTG-3′; Reverse primer: 5′-GGGCTGCAGAATCTAGAGCGGCCG CCTTTTTTTTTTTT-3′) and ligated into the vector by Gibson assembly (NEB Gibson Assembly cloning kit E5510S). The CYFIP2mutE was obtained by mutagenesis of the 727K-E from CYFIP2WT with the QuikChange II Site-Directed Mutagenesis Kit. Human *cyfip1* cDNA was obtained from dharmcon/GE Healthcare (MHS678, ID 3163591). Human *cyfip2* cDNA was obtained from Source Bioscience (IRAUp969A0861D, ID 3619680). The pCS2-Cas9 plasmid was originally from the Schier lab (addgene, 47322). All plasmids were verified by sequencing. mRNAs were produced from linearized plasmids by using the SP6 mMessage Machine kit (AM1340), cleaned using the QIAGEN RNeasy kit, and the purity and concentrations were finally measured by nanodrop spectrophotometer. Morpholinos (MOs) were obtained from Gene Tools. *Xenopus* CYFIP2 (TTACCAAGTCCGGTAGCGACAGTCT) and control MOs were conjugated to FITC and injected at 10 ng each. To block *cyfip2* splicing in zebrafish, the following morpholino was used (Pittman et al., 2010): zCYFIP2– AGTGCATTAGGACGTGTACCTGGTA, 3 ng.

gRNAs design and quantification of CRISPR efficiency

Guidance RNAs (gRNAs) were designed by using the design tool at http://chopchop.cbu.uib.no. The specific sequences for the gRNA*cyfip1* exon 2 (GGAGGGCAGAGGCTCGATGC) and gRNA*cyfip2* exon 2 (GACAACCCACGTGACCCTGG) were selected. For the generation of the gRNAs and *cas9* mRNAs, we followed the previously established protocol in (Shah et al., 2015). Briefly, we generated a specific guide-template PCR product for each gRNA and 1 μg was used for T7 *in vitro* transcription reaction (MEGAscript T7, Life Technologies). gRNAs were then purified by column (Zymo Research, R1016) and a nanodrop spectrophotometer was used to check the purity and concentrations. All injections contained 1,200 ng/μL of Cas9-encoding mRNA. sgRNAs for *cyfip1* and *cyfip2* were diluted to 200 ng/μL. To analyze the CRISPR efficiency, we isolated DNA from 10 F_0 embryos (Phire animal tissue direct PCR kit, ThermoFisher) and specific primers were used to amplify the target cut site. The PCR products were then purified using an RNeasy mini kit (QIAGEN) and analyzed by sequencing.

Xenopus and zebrafish embryo injection

Xenopus embryos injections were performed at four-cell stage in both dorsal blastomeres. Embryos were de-jellied with 2% cysteine (Sigma) in 1X MBS (pH 8), rinsed 3x in 0.1X MBS and aligned on a grid in 4% Ficoll (Sigma) in 0.1X MBS, 1% penicillin (100 U/ml), streptomycin (100 μg/mL) and fungizone 0.25 μg/mL (PFS, GIBCO). Injections of 5 nL of volume were performed using glass capillary needles (1.0 mm OD x 0.5 mm, Harvard Aparatus) and a microinjector (Picospritzer, General Valve). Zebrafish injections were made at one cell stage into the yolk (morpholinos) or the cell (CRISPR). Injections were performed using 0.78mm needles pulled with a needle puller (1.0 mm OD x 0.78 mm, Harvard Asparatus; puller: Pul-1, World Precision Instrument) and 1 nL of volume was pressure injected using an air-pressure injector (Picospritzer II, Intracel).

Lipophilic dye labeling and imaging

Zebrafish embryos were fixed with 4% paraformaldehyde (PFA) at 48 hpf or 5 dpf, and kept at 4°C for 24 hr. Embryos were then pinned down on a silgar plate in 1x PBS and the Dorsal (D) or Ventral (V) quadrants of the retina were injected using a microinjector (Picospritzer, General Valve) and 0.5 mm needles pulled. D retina was injected with 1,1'-dioctadecyl-3,3,3′,3′-tetramethylindocarbocyanine perchlorate (DiI, Molecular Probes) dissolved in 100% ethanol (Sigma), and V retina with 3,3′-dioctadecyloxacarbocyanine perchlorate (DiO, Molecular Probes) dissolved in dimethylformamide (DMF, Sigma), and incubated during 24 hr at RT. Both eyes were then removed using dissection pins and whole-mount embryos were mounted laterally in agarose (24–28°C gelling point, Promega). Images were acquired using a Perkin Elmer Spinning Disk UltraVIEW ERS, Olympus IX81 inverted spinning disk confocal microscope with 30x silicon immersion objective and Volocity imaging software (Perkin Elmer). Images are shown as maximal projections of z series.

Electroporation and *in vivo* analysis of axon-axon interactions

Targeted electroporation was carried out as previously described (Falk et al., 2007). At stage 28, *Xenopus* embryos were anesthetized with 40 mg/100 mL MS222 in 1X MBS, followed by injection of pCS2+mGFP cDNA (2 μg/μL) into the ventricle between the retina and the brain. Four electric pulses of 50 ms duration were delivered at 18V and 1000 ms intervals. The embryos were then recovered and raised in 0.1X MBS. At stage 35/36 or 37/38, embryos were anesthetized with 40 mg/100 mL MS222 in 1X MBS. On the contralateral hemisphere of the electroporated eye, the lateral surface of the optic tract was exposed by carefully removing the overlying eye and skin (Chien et al., 1993). The embryos were then recovered in 10 mg/100 mL MS222 in 1X MBS and mounted into an oxygen-permeable chamber consist of a Permanox slide (Sigma-Aldrich) and Gene Frame (Thermo Scientific). Time-lapse imaging at 30 s per frame was performed at 60X with Perkin Elmer Spinning Disk UltraVIEW ERS, Olympus IX81 inverted spinning disk confocal microscope. Z stack intervals of 1.5 μm were used for acquiring images with Volocity (Improvision).

Xenopus Retinal explant cultures

50 mm glass-bottom dishes (Matek) were coated with poly-L-lysine (PLL; 10 μg/mL) diluted in double distilled H2O (ddH2O) for a minimum of 3 hr at room temperature (RT). PLL was washed 3 times with ddH2O, followed by coating with laminin (10 μg/mL, Sigma) in L-15 medium (GIBCO) for 1 hr at RT. Embryos stage 33/34 were washed 3 times in 0.1X MBS with 1% PFS to remove bacteria. Embryos were anesthetized with MS222 and aligned on a sylgard-coated dish in 60% L-15 culture medium (60% L-15 in ddH2O and

1% PFS and MS222, pH 7.6-7.8). Anesthetized embryos were secured on their lateral side with custom made pins and the eye dissected out using dissection pins. Whole eye, Dorsal or Ventral eye pieces were then washed in 60% L-15 and plated on pre-coated dishes containing 60% L-15 culture medium (60% L-15 medium supplemented with 1% PFS). Dishes were incubated at 20°C for 12-24 hr depending on the experiment.

Live imaging in *Xenopus* retinal ganglion cell axons

Xenopus embryos were injected with 200 pg of DNA encoding CYFIP2-GFP, CYFIP2ΔCTD-GFP or CYFIP2mutE-GFP per blastomere. For labeling RNP granules, *Xenopus* embryos were injected with 5 nL of 100 μM Cyanine 3-UTP (Perkin Elmer). Stage 24/25 embryos with positive fluorescence labeling in the eye primordia were selected for culture the following day. Culture medium was replaced for imaging (60% Phenol Red-free L-15 medium supplemented with 1% PFS). To visualize the growth cone cell membrane, the Cell-Vue Maroon Labeling Kit was used (ThermoFisher Scientific). RGC axons were imaged on a Perkin Elmer Spinning Disk UltraVIEW ERS, Olympus IX81 Inverted microscope with 60x silicon immersion objective and Volocity imaging software (Perkin Elmer).

Pharmacological treatments

The following pharmacological agents were bath applied to retinal axon-only cultures as indicated: 50 μM cycloheximide (Sigma), 50 nM rapamycin (Calbiochem), 50 nM Cytochalasin D (Sigma), or Dimethyl sulfoxide as a control (Sigma). For zebrafish treatment, the following agents were added to the embryo medium from 24 hpf and replaced every 24 hr: 500 nM rapamycin (Calbiochem) and Dimethyl sulfoxide (DMSO) as a control (Sigma).

Immunostainings and proximity ligation assay

Eye explant cultures from *Xenopus* were fixed in 4% PFA and 15% sucrose in 1X PBS for 20 min, washed 3 times 10 min in 1X PBS and permeabilized for 3 min in 0.1% triton (Sigma) in 1X PBS. The explants were washed 3 more times in 1X PBS and blocked for 60 min in blocking mix (5% heat inactivated horse serum (HIHS) in 1X PBS). Primary antibodies were diluted in blocking mix and added to the explants for 24 hr at 4°C. The explants were then washed 3 times 10 min in PBS before incubation with the secondary antibody in blocking mix for 60 min at RT. Phalloidin-alexa658 (Life Technologies) was diluted in blocking mix and added to the explant for 1 hr. The explants were washed a final 3 times in 1X PBS before mounted with FluorSave reagent (Calbiochem). For proximity ligation assay (PLA), the same protocol was applied as previously mentioned for primary antibodies followed by PLA protocol as described by the manufacturer (Duolink *in situ* kit, SIGMA).

For immunostaining on cryostat sections, zebrafish embryos were fixed for overnight in 4% PFA at 4°C, rinsed 3x in 1x PBS and put in 30% sucrose in 1x PBS for a minimum of 30 min. Embryos were embedded in Tissue-TEK OCT compound (SAKURA) and quick frozen on dry ice or at −80°C. Transverse sections with a 20 μm thickness were cut using a cryostat (Leica CM3050S). Slides were washed 3x 10 min in PBS and blocked for 2 hr in blocking buffer (5% HIHS, 0.1% Triton, 1x PBS). Primary antibodies in blocking buffer were incubated over night (O/N) at RT in a humidified chamber. Slides were then washed 3x in 1x PBS and incubated with secondary antibody in blocking buffer for 1 hr at RT in a humidified chamber in the dark. Slides were washed a final 3x 10 min in PBS, incubated with 1:10000 DAPI for 45 min in a humidified chamber at RT, drained of and mounted with FluorSave reagent. All slides were imaged using a Perkin Elmer Spinning Disk UltraVIEW ERS, Olympus IX81 Inverted microscope and 20x (0.45 NA) objective.

The following primary antibodies were used: anti-CYFIP2 (ab79716, Abcam), anti-CYFIP1 (ab108220, Abcam), anti-NCKAP1 (ab140856, Abcam), anti-xFXR (gift from Dr. Khandjian).

Immunoprecipitation and puromycin assay

Xenopus brains and eyes were dissected from stage 35/36 embryos and homogenized in lysis buffer (Tris 20 mM, NaCl 100 mM, 1% NP40, 10% Glycerol, 10 mM MgCl2) supplemented with Halt protease and phosphatase inhibitor cocktail (Invitrogen) during 30 min at 4°C. Following centrifugation, the supernatant was collected. For immunoprecipitation, the protein extracts were incubated overnight at 4°C with the indicated antibody pre-conjugated to Protein G-magnetic beads (Dynabeads Protein G, Life Technologies 10004D). Fish heads were dissected at 72 hpf and homogenized in lysis buffer. For the puromycin assay, 24 hpf zebrafish embryos were incubated in E3 embryo medium containing 200 μg/mL of puromycin (Sigma). Fish heads were dissected at 48hpf and homogenized in RIPA buffer (Sigma) supplemented with Halt protease and phosphatase inhibitor cocktail (Invitrogen). For western blot analysis, proteins were resolved by 10% SDS-PAGE and transferred to nitrocellulose membrane (Bio-Rad). The following primary antibodies were used: anti-CYFIP2 (1:500, ab79716, Abcam), anti-NCKAP1 (1:500, ab140856, Abcam), anti-xFXR (1:1000, gift from Dr. Khandjian), anti-ELAVLs (1:500, sc-5261, Santa Cruz), anti-rpl10a (1:500, 16681-1-AP, Proteintech), anti-rps3A (1:500, 14123-1-AP, Proteintech), anti-puromycin (1:1000, Milipore) and anti-tubulin (1:10000, Millipore). Bands were then detected using an ECL-based detection (GE Healthcare).

QUANTIFICATIONS AND STATISTICAL ANALYSIS

Statistics

Data were analyzed with PRISM 6 (GraphPad) and the statistical test used for each figure is reported in the corresponding figure legend. *$p < 0.05$, **$p < 0.01$, ***$p < 0.001$, n.s: non-significant. Data are usually presented as mean, unless mentioned in the figure

legend, and errors bars always represent SEM. The “n” are indicated on the graph, and what “n” represent is reported in the corresponding figure legend. The number of experiment is indicated in the figure legends or text.

In vivo axonal projection and Missorting index

Maximum projections images were imported in ImageJ for quantifications. Quantifications of axonal projections of V and D axons at 48hpf were obtained by creating a mask for each signal and measuring the area. The method to generate the “missorting index” is the same as previously used by Chien and colleagues (Poulain and Chien, 2013). A line of the size of the total tract width (D+V axons) was drawn perpendicular to the tract at 70 μm from the point of origin of the axons from the optic chiasm. The mean intensity of the DiI signal (dorsal axons) along this line was measured in each brachia of the tract for each embryo. The missorting index was then calculated as the ratio of the fluorescence signal intensity corresponding to missorted dorsal axons (Dm) to the signal intensity of all dorsal axons (properly sorted = Ds + missorted = Dm). This quantification assumes a correlation between mean signal intensity and amount of axons labeled. We therefore obtained the relative amount of signal from missorted dorsal axons compare to the total amount of signal from dorsal axons in the optic tract for each embryo.

In vivo analysis of axon-axon interactions

Spontaneous axon-axon interaction events were identified by manually going through the z stacks. The navigational motions of the identified axons were determined by tracking through time. These motions were categorized into crossing, tracking, and fasciculation events, which were assessed statistically with Fisher’s exact test.

Axon-axon interaction assay

Dorsal or Ventral retina explants from stage 33/34 *Xenopus* embryos expressing gap-RFP or gap-GFP were incubated for 12-36 hr before imaging. Thanks to the fluorescent expression, the origin of the axon was ascertained by eye and bright-field time-lapse imaging was started before the growth cone made contact with the axon shaft and terminated at the end of a behavior or after 45 min had passed. Images were taken every 30 s using the Hamamatsu photonics camera on the Nikon Eclipse TE2000-U inverted microscope and Volocity imaging software. Images were analyzed using Fiji (ImageJ). The axonal behaviors were categorized into fasciculation (an axon is determined as fasciculating if its axon shaft merges with the encounter axon), crossing (an axon is determined as crossing if its axon shaft and growth cone crosses the encounter axon), tracking (an axon is determined as tracking if we observe multiple growth cone filopodia contacts with the encounter axon leading to a change in direction) and stalling/retraction events (an axon is determined as stalled if it neither moves forward nor retracts after 45 min following the contact). In order to perform a quantitative analysis, the results from a large number of independent experiments for each condition were pooled, which were assessed statistically with Fisher’s exact test.

CYFIP2-GFP and RNA dynamics

Volocity software was used for analysis. CYFIP2-GFP and Cy3-labeled RNA co-localizing granules were tracked over 1 min and classified as anterograde or retrograde transport if the granule displace more than 2 μm in one direction from their origin. Otherwise the granule was classified as static/oscillatory. For Cy3-RNA granules dynamics in GC, a first snapshot phase contrast image was taken (T = 0), followed by 10 min recording at maximum speed using the Hamamatsu photonics camera on the Nikon Eclipse TE2000-U inverted microscope and Volocity imaging software. For the analysis, the growth cone outline and domains were traced on the phase contrast image using Volocity and then superimposed on the fluorescent images. Cy3-RNA granules were manually counted every 30 s. For the anterograde transport analysis, the number of Cy3-RNA granules displaying anterograde transport in the 50 μm segment proximal to, and reaching, the growth cone was manually counted over the 10 min recording. For presentation clarity, images in Figures 4B, 4C, 6E, and 7B were denoised with ND-SAFIR (Boulanger et al., 2010).

Analysis of filopodia dynamics

Phase contrast time-lapse sequences of growth cones in each condition were acquired using Volocity on a Nikon Eclipse 80i microscope (60X objective) with Hamamatsu ORCA-ER camera at 1frame/second during 5 min. Projection from the growth cone periphery equal to or longer than 2 μm was considered as a filopodium. The number of filopodia per growth cone and the lengths of filopodia were measured manually using Volocity software on fixed cultures in Figures 4I and 4J or at the first frame in Figures S2C and S2D. Analysis of filopodia dynamics was performed over 5 min recording. Filopodia were defined as “formation” if they were newly generated, “retraction” if they were completely retracted and “stable” if they were present throughout the 5 min. Analysis of filopodial elongation and retraction speeds and lifetime were performed using Volocity software. The filopodial tip was tracked manually every 5 s until the maximum length for elongation or less than 2 μm for retraction. The filopodia elongation or retraction speed was then calculated in between consecutive measurements and only the values during active phase of filopodia movement were used for each filopodium. The time during which the filopodium remained above 2 μm was considered as the filopodial lifetime.

Immunocytochemistry and PLA analysis

For the quantification of fluorescence intensity, the axon or the growth cone (global, central, and peripheral domains) outlines were traced on the phase contrast image using Volocity (PerkinElmer) and then superimposed on the fluorescent image. The software then calculated the pixel intensity per unit area within the analyzed area. The same outline was then placed in an adjacent area to record the background fluorescent intensity. This value was subtracted from the growth cone reading, providing the background-corrected intensity of the signal. For the colocalization analysis, Mender's coefficient was calculated using coloc2 plugin in Fiji (ImageJ).

For the quantification of the PLA signal, the axon or growth cone central and peripheral domains were traced on the phase contrast image using Volocity and the number of dots counted manually in the respective areas.

Neuron, Volume *97*

Supplemental Information

Axon-Axon Interactions Regulate

Topographic Optic Tract Sorting

via CYFIP2-Dependent WAVE Complex Function

Jean-Michel Cioni, Hovy Ho-Wai Wong, Dario Bressan, Lay Kodama, William A. Harris, and Christine E. Holt

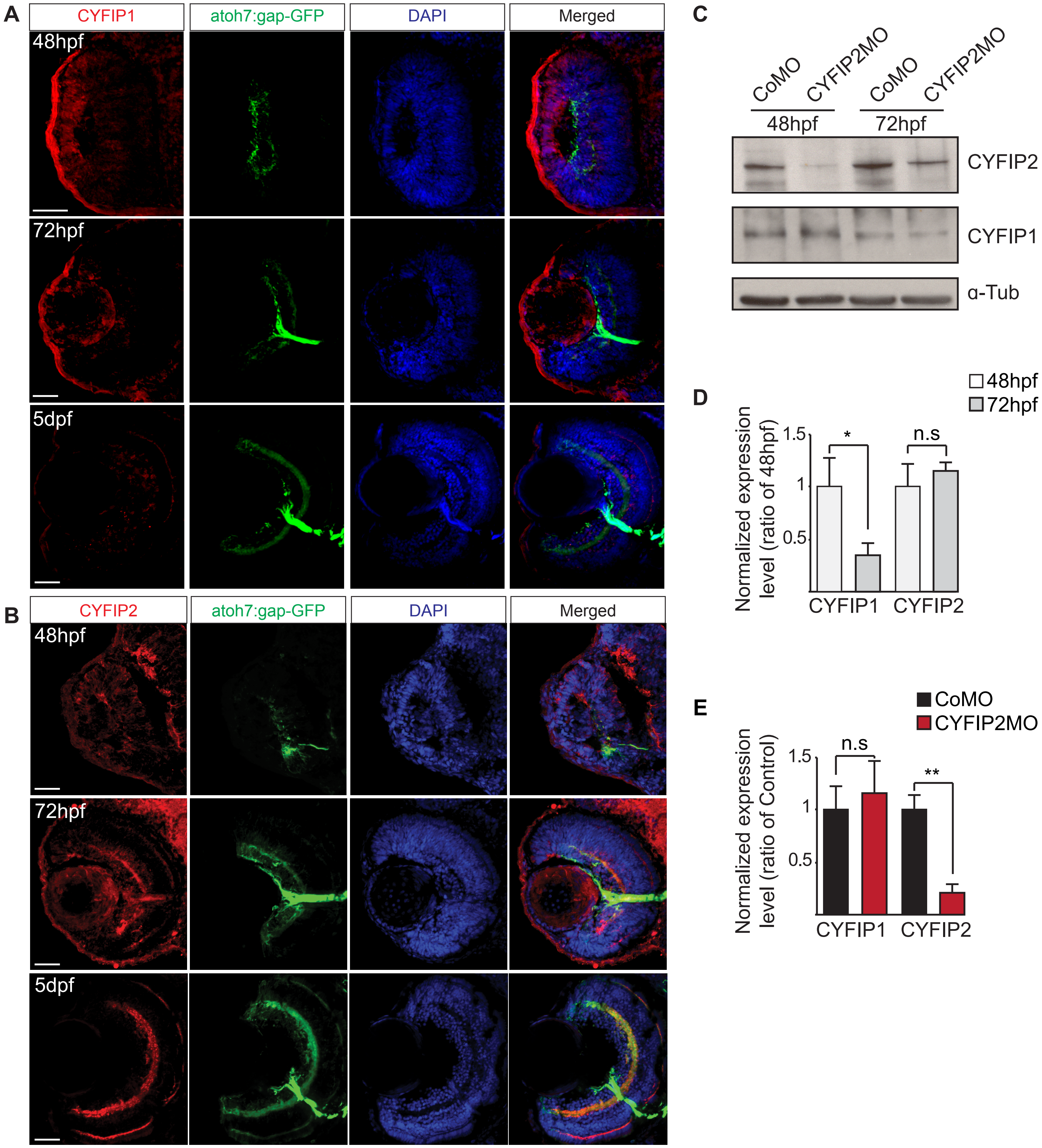
A
CYFIP1
atoh7:gap-GFP
DAPI
Merged
48hpf
72hpf
5dpf
B
CYFIP2
atoh7:gap-GFP
DAPI
Merged
48hpf
72hpf
5dpf
C
CoMO
CYFIP2MO
CoMO
CYFIP2MO
48hpf
72hpf
CYFIP2
CYFIP1
α-Tub
D
48hpf
72hpf
Normalized expression level (ratio of 48hpf)
1.5
1
0.5
*
n.s
CYFIP1
CYFIP2
E
CoMO
CYFIP2MO
Normalized expression level (ratio of Control)
1.5
1
0.5
n.s
**
CYFIP1
CYFIP2

FIGURE S1

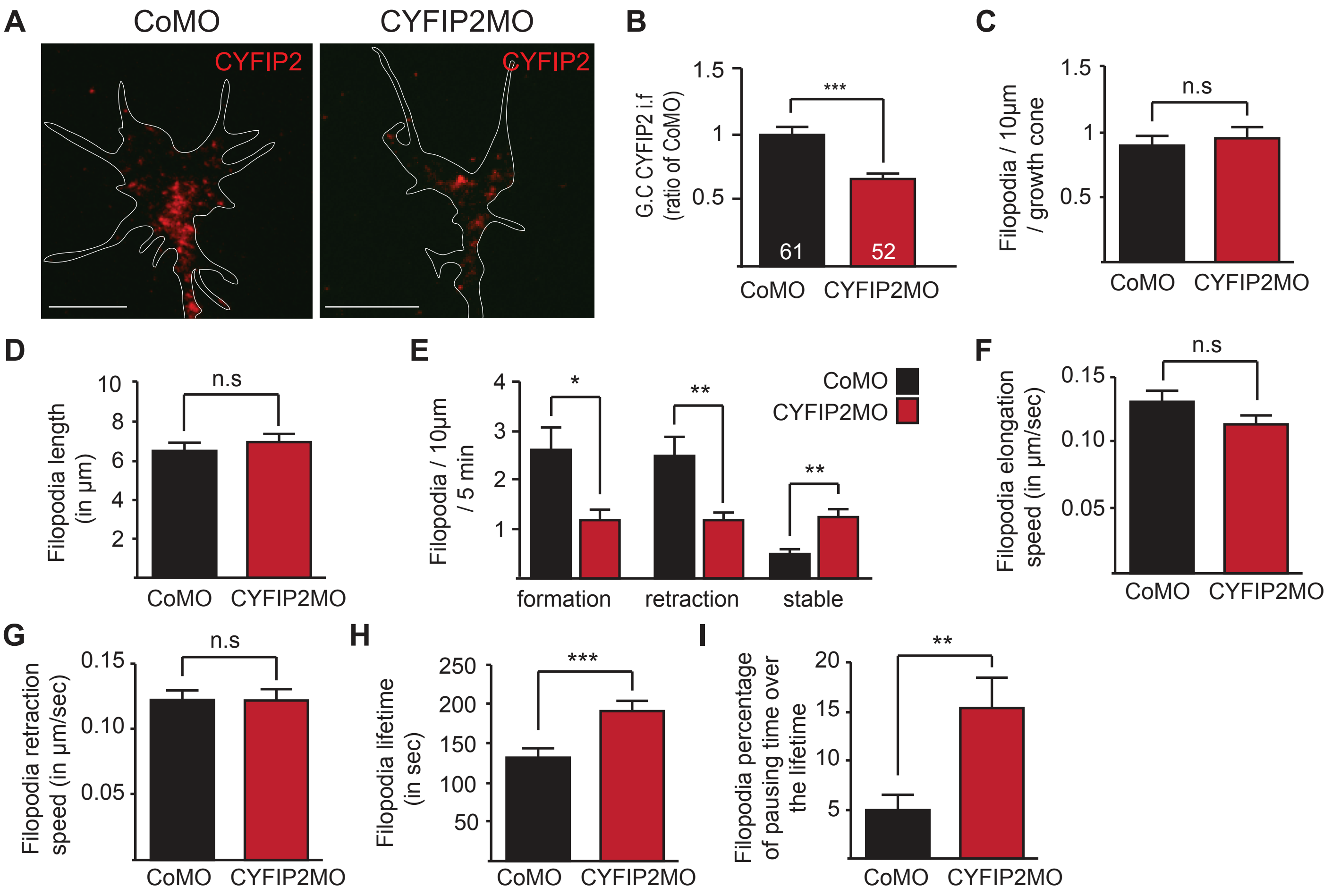
A
CoMO
CYFIP2MO
CYFIP2
CYFIP2
B
G.C CYFIP2 i.f (ratio of CoMO)
1.5
1
0.5
***
61
52
CoMO
CYFIP2MO
C
Filopodia / 10µm / growth cone
1.5
1
0.5
n.s
CoMO
CYFIP2MO
D
Filopodia length (in µm)
10
8
6
4
2
n.s
CoMO
CYFIP2MO
E
Filopodia / 10µm / 5 min
4
3
2
1
*
**
**
CoMO
CYFIP2MO
formation
retraction
stable
F
Filopodia elongation speed (in µm/sec)
0.15
0.10
0.05
n.s
CoMO
CYFIP2MO
G
Filopodia retraction speed (in µm/sec)
0.15
0.10
0.05
n.s
CoMO
CYFIP2MO
H
Filopodia lifetime (in sec)
250
200
150
100
50
***
CoMO
CYFIP2MO
I
Filopodia percentage of pausing time over the lifetime
20
15
10
5
**
CoMO
CYFIP2MO

FIGURE S2

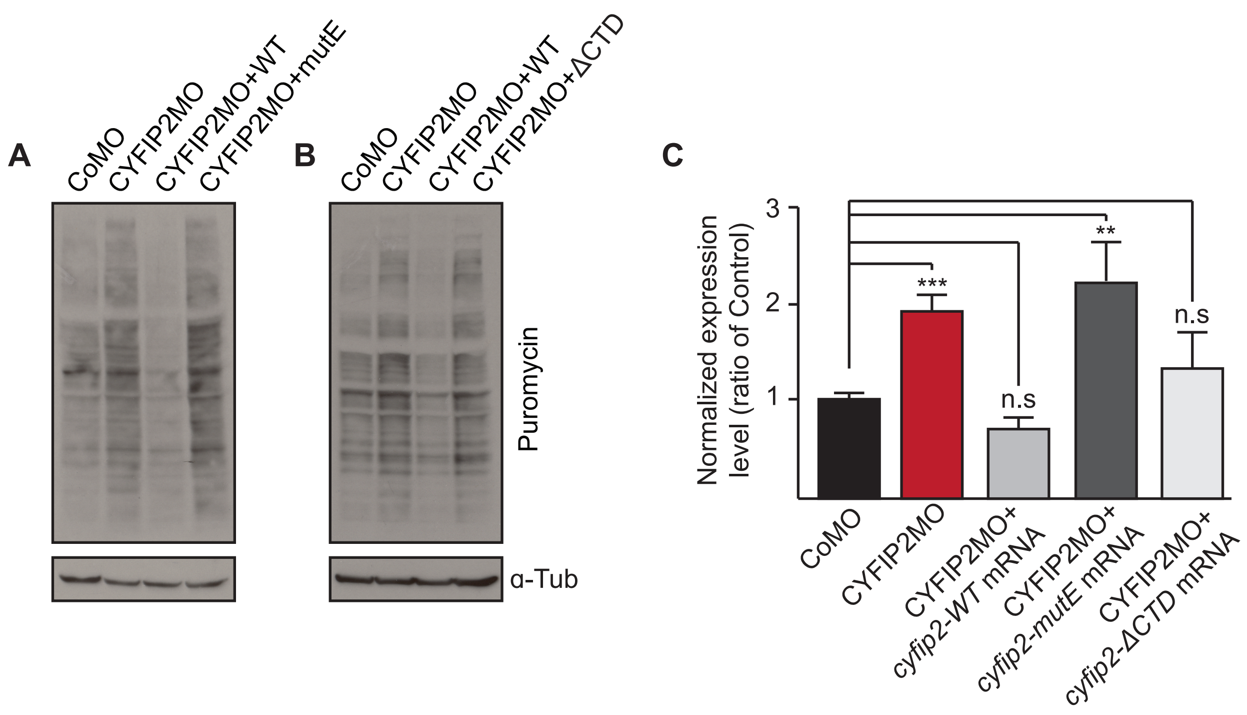
A
CoMO
CYFIP2MO
CYFIP2MO+WT
CYFIP2MO+mutE
B
CoMO
CYFIP2MO
CYFIP2MO+WT
CYFIP2MO+ΔCTD
Puromycin
α-Tub
C
Normalized expression level (ratio of Control)
3
2
1
***
n.s
**
n.s
CoMO
CYFIP2MO
CYFIP2MO+ cyfip2-WT mRNA
CYFIP2MO+ cyfip2-mutE mRNA
CYFIP2MO+ cyfip2-ΔCTD mRNA

FIGURE S3

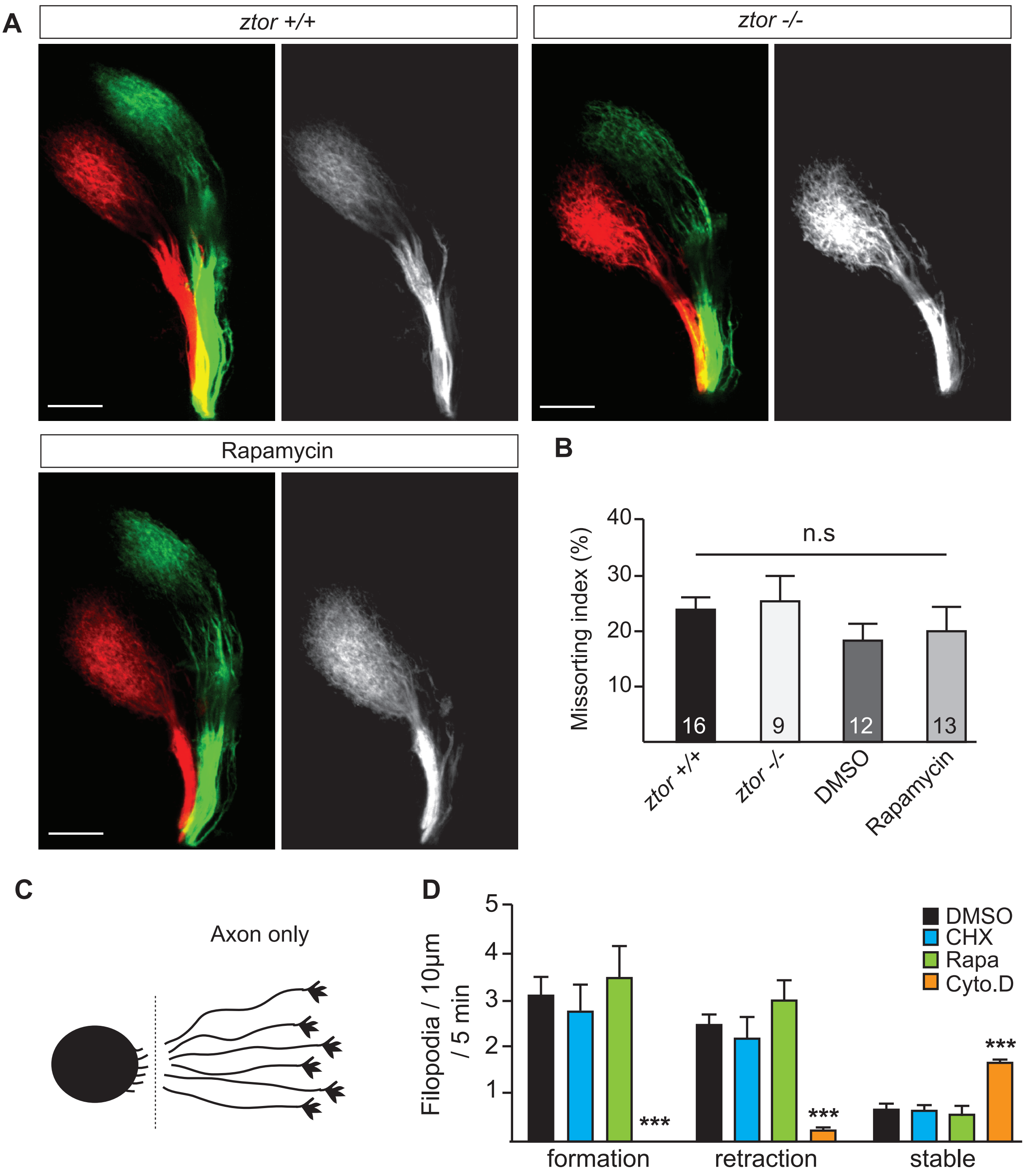
A
ztor +/+
ztor -/-
Rapamycin
B
n.s
Missorting index (%)
40
30
20
10
16
9
12
13
ztor +/+
ztor -/-
DMSO
Rapamycin
C
Axon only
D
Filopodia / 10µm / 5 min
5
4
3
2
1
***
***
***
formation
retraction
stable
DMSO
CHX
Rapa
Cyto.D

FIGURE S4

Supplementary information

Figure S1: Related to Figure 1. CYFIP1 and CYFIP2 expression during early zebrafish retina development.

(A) Representative immunostainings of CYFIP1 and DAPI at 48hpf, 72hpf and 5dpf on retina slices from atoh7:gap-GFP zebrafish line (n=8 zebrafish for each developmental stage). Scale bars=50µm. (B) Representative immunostainings of CYFIP2 and DAPI at 48hpf, 72hpf and 5dpf on retina slices from atoh7:gap-GFP zebrafish line (n=12 zebrafish for each developmental stage). Scale bars=50µm. (C-E) Representative western blots for CYFIP1 and CYFIP2 on zebrafish heads lysates at 48hpf and 72hpf for CoMO- and CYFIP2MO-injected embryos. (D) Quantifications of the signal obtained for CYFIP1 and CYFIP2 at 48hpf compared to 72hpf (n=4, normalized to α-Tubulin, Mann Whitney test). (E) Quantifications of the signal obtained for CYFIP1 and CYFIP2 at 48hpf in CoMO and CYFIP2MO conditions (n=4, normalized to α-Tubulin). Error bars represent SEM. *p<0.05, **p<0.01, n.s: non-significant (Mann Whitney test for D and E).

Figure S2: Related to Figure 4. CYFIP2 regulates the dynamic behaviors of filopodia in RGC growth cones.

(A) Representative CYFIP2 immunostainings on stage 32 Xenopus retinal growth cone (GC) in CoMO and CYFIP2MO conditions. (B) Quantification of CYFIP2 signal intensity in GC of CYFIP2MO- compared to CoMO-injected embryos. Number of axons analyzed is indicated on the graph. (C, D) Quantifications of the number (C) and length (D) of GC filopodia in CYFIP2MO (n=18 GC, n=101 filopodia) compared to CoMO (CoMO: n=16 GC, n=80 filopodia) conditions. (E) Quantifications of the frequency of formation, retraction and stable filopodia in GCs from CYFIP2MO-injected (n=17 GC) compared to CoMO-injected (n=15 GCs) retina explants over 5min recording. (F) Quantifications of the speed during active filopodia elongation in CYFIP2MO (n=15 filopodia) compared to CoMO (n=15 filopodia) conditions. (G) Quantifications of the speed during active filopodia retraction in CYFIP2MO

(n=13 filopodia) compared to CoMO (n=10 filopodia) conditions. (H) Quantifications of filopodia lifetime in CYFIP2MO (n=13 GC, n=97 filopodia) compared to CoMO (n=11 GC, n=111 filopodia) conditions. (I) Percentage of the time spent pausing during the filopodia lifetime. Error bars represent SEM. * $p<0.05$, ** $p<0.01$, *** $p<0.001$, n.s: non-significant (Mann Whitney test for B-I). Scale bars 5μm.

Figure S3: Related to Figure 7. CYFIP2 exerts a translational control *in vivo*

(A-B) Representative western blots showing puromycin incorporation in 48hpf zebrafish embryos for the indicated conditions. (C) Quantification of the signal obtained for puromycin normalized to α-Tubulin. Co-injection of CYFIP2MO + *CYFIP2WT* (n=7experiments) or *CYFIP2ΔCTD* (n=4 experiments), but not *CYFIP2mutE* (n=5 experiments), mRNAs rescue the increase in puromycin signal observed in CYFIP2MO-inected embryos (n=7 experiments) compared to CoMO (n=7 experiments). Error bars represent SEM. ** $p<0.01$, *** $p<0.001$, n.s: non-significant (Mann Whitney test).

Figure S4: Related to Figure 7. Translation regulation is not required for proper growth cone filopodial dynamics and D-V axon sorting.

(A) Dorsal and Ventral RGC axons were labeled by respective injections of DiI (red) and DiO (green) fluorescent dyes in zebrafish embryos retina at 5dpf. Scale bars: 50 μm. (B) The missorting index (MI) was quantified as the ratio of the intensity signal of the missorted D (Dm) axons to all the D axons (Dm+Ds) (Mann Whitney test, n.s: non significant). The number of zebrafish analyzed is indicated on the bars. (C) Schematic illustrating the axon-only culture used for the analysis of filopodia dynamics. (D) Quantifications of the frequency of formation, retraction and stable filopodia in growth cones of axon-only explants treated with DMSO (n=7 GC), cycloheximide (CHX) (n=7 GC), rapamycin (rapa) (n=6 GC) and cytochalasin D (cyto.D) (n=7 GC). Error bars represent SEM. *** $p<0.001$ (Mann Whitney test for B and D).
